# Cancer‐associated V‐ATPase induces delayed apoptosis of protumorigenic neutrophils

**DOI:** 10.1002/1878-0261.12630

**Published:** 2020-01-31

**Authors:** Safaa A. Ibrahim, Arpita Kulshrestha, Gajendra K. Katara, Valerie Riehl, Manoranjan Sahoo, Kenneth D. Beaman

**Affiliations:** ^1^ Department of Microbiology and Immunology Faculty of Pharmacy Cairo University Egypt; ^2^ Department of Microbiology and Immunology Rosalind Franklin University of Medicine and Science North Chicago IL USA

**Keywords:** cytokines, NF‐κB, TLR2, tumor microenvironment, tumor‐associated neutrophils, vacuolar ATPase

## Abstract

Tumors and neutrophils undergo an unexpected interaction, in which products released by tumor cells interact to support neutrophils that in turn support cancer growth, angiogenesis, and metastasis. A key protein that is highly expressed by cancer cells in tumors is the a2 isoform V‐ATPase (a2V). A peptide from a2V (a2NTD) is secreted specifically by cancer cells, but not normal cells, into the tumor microenvironment. This peptide reprograms neutrophils to promote angiogenesis, cancer cell invasiveness, and neutrophil recruitment. Here, we provide evidence that cancer‐associated a2V regulates the life span of protumorigenic neutrophils by influencing the intrinsic pathway of apoptosis. Immunohistochemical analysis of human cancer tissue sections collected from four different organs shows that levels of a2NTD and neutrophil counts are increased in cancer compared with normal tissues. Significant increases in neutrophil counts were present in both poorly and moderately differentiated tumors. In addition, there is a positive correlation between the number of neutrophils and a2NTD expression. Human neutrophils treated with recombinant a2NTD show significantly delayed apoptosis, and such prolonged survival was dependent on NF‐κB activation and ROS generation. Induction of antiapoptotic protein expression (Bcl‐xL and Bcl‐2A1) and decreased expression of proapoptotic proteins (Bax, Apaf‐1, caspase‐3, caspase‐6, and caspase‐7) were a hallmark of these treated neutrophils. Autocrine secretion of prosurvival cytokines of TNF‐α and IL‐8 by treated neutrophils prolongs their survival. Our findings highlight the important role of cancer‐associated a2V in regulating protumorigenic innate immunity, identifying a2V as a potential important target for cancer therapy.

## Graphical abstract

Tumors and neutrophils undergo an unexpected interaction. A key protein that is highly expressed by cancer cells in tumors is the a2 isoform V‐ATPase (a2V). A peptide from a2V (a2NTD) is secreted specifically by cancer cells into the tumor microenvironment. This peptide reprograms neutrophils to support their survival and promotes its protumorigenic properties, and neutrophils in turn support tumor aggressiveness.

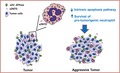

**Abbreviations**
a2NTD: N‐terminal domain of a2V
a2NuΦ: a2NTD‐treated neutrophils
a2V: a2 isoform of the V0 a‐subunit of V‐ATPase
Apaf‐1: apoptotic protease‐activating factor 1
Bax: Bcl‐2‐associated X protein
Bcl‐2A1: Bcl‐2‐related protein A1
Bcl‐xL: B‐cell lymphoma‐extra large
BSA: bovine serum albumin
c‐FLIP: cellular FLICE (FADD‐like IL‐1β‐converting enzyme) inhibitory protein
Cl. PARP: cleaved poly (ADP‐ribose) polymerase
Cyt‐*c*: cytochrome *c*
FasL: Fas ligand
FBS: fetal bovine serum
G‐CSF: granulocyte‐colony stimulating factor
GM‐CSF: granulocyte‐macrophage colony‐stimulating factor
HMGB1: high mobility group box 1
HSPs: heat‐shock proteins
IL‐8: interleukin‐8
LDN: low‐density neutrophil
LPS: lipopolysaccharide
LUAD: human lung adenocarcinoma
Mcl‐1: myeloid cell leukemia 1
MDSC: myeloid‐derived suppressor cells
MFI: mean fluorescence intensity
MMP: mitochondrial membrane potential
MMP‐9: matrix metallopeptidase 9
NAC: N‐acetyl‐L‐cysteine
NF‐κB: nuclear factor kappa‐light‐chain‐enhancer of activated B cells
PARP: poly (ADP‐ribose) polymerase
PRR: pattern recognition receptor
qRT‐PCR: quantitative real‐time PCR
R.I.: reciprocal intensity
ROS: reactive oxygen species
TAN: tumor‐associated neutrophil
TCGA: The Cancer Genome Atlas
TIMER: Tumor IMmune Estimation Resource
TLR2: Toll‐like receptor‐2
TME: tumor microenvironment
TNF‐α: tumor necrosis factor alpha
TOM20: translocase of the outer membrane 20
V‐ATPases: vacuolar ATPases
VEGF: vascular endothelial growth factor

## 1 Introduction

The complex interactions in the tumor microenvironment between malignant cells and immune cells are crucial for tumor establishment and metastasis (Coffelt *et al.*, 2016; Mantovani, 2014; Mantovani *et al.*, 2008; Veglia *et al.*, 2018). Tumor‐associated neutrophils (TANs) represent a significant portion of tumor‐infiltrating leukocytes and are key cells promoting tumor progression (Clappaert *et al.*, 2018; Mantovani, 2014; Mollinedo, 2019; Treffers *et al.*, 2016; Whyte *et al.*, 2018). Tumor microenvironment (TME) reprograms TAN to promote the secretion of protumorigenic factors including reactive oxygen species, arginase‐1, IL‐8, VEGF, and MMP‐9 (Clappaert *et al.*, 2018; Fridlender *et al.*, 2009; Mollinedo, 2019; Powell and Huttenlocher, 2016; Whyte *et al.*, 2018). TAN‐secreted factors contribute to each stage of cancer, from initiation to metastasis and development of resistance to cancer therapy (Clappaert *et al.*, 2018; Coffelt *et al.*, 2016; Zhang *et al.*, 2016). TME also promotes neutrophil recruitment and survival despite the known fact that they have short life span in blood (Fridlender and Albelda, 2012; Kumar *et al.*, 2016; Powell and Huttenlocher, 2016). Understanding the mechanisms that cancer‐associated factors regulate neutrophil survival and their protumorigenic properties can be used to develop specific cancer therapy.

Vacuolar ATPases are multisubunit proton pumps, important for normal physiological homeostasis (Beaman *et al.*, 2016; Katara *et al.*, 2018; Sahoo *et al.*, 2018; Stransky *et al.*, 2016). However in cancer, uniquely specific subunits of V‐ATPases are highly expressed by tumor cells and can be novel targets for cancer therapy (Di Cristofori *et al.*, 2015; Kulshrestha *et al.*, 2016, 2019; Stransky *et al.*, 2016; Whitton *et al.*, 2018). The a2 isoform of the V0 a‐subunit of V‐ATPase (a2V) is highly expressed in breast (Ibrahim *et al.*, 2015; Katara *et al.*, 2014; Pamarthy *et al.*, 2015) and ovarian (Kulshrestha *et al.*, 2015, 2016) cancer, specifically on the cell membrane of cancer cells (Ibrahim *et al.*, 2015; Kulshrestha *et al.*, 2015; Pamarthy *et al.*, 2015). Microvesicles secreted from cancer cells contain a peptide from the N‐terminal domain of a2V (a2NTD), and this peptide is not detected in microvesicles secreted by normal cells (Ibrahim *et al.*, 2015; Katara *et al.*, 2014; Kwong *et al.*, 2011). a2NTD has important immunoregulatory properties in the tumor microenvironment (Ibrahim *et al.*, 2015, 2016; Katara *et al.*, 2014), it promotes the protumorigenic characteristics of neutrophils and macrophages that enhance tumor angiogenesis and cancer cell invasion (Ibrahim *et al.*, 2015, 2016; Katara *et al.*, 2014; Kwong *et al.*, 2011). Knocking down a2V in 4T1 cells decreases a2NTD secretion and delays tumor growth *in vivo* due to the decrease in the protumorigenic macrophage population (Katara *et al.*, 2015). Also, a2NTD increases neutrophil recruitment to the site of the tumor (Ibrahim *et al.*, 2016). The facts that protumorigenic TANs have long life span (Andzinski *et al.*, 2015; Fridlender and Albelda, 2012; Pylaeva *et al.*, 2016) and a2NTD enhances the protumorigenic properties of neutrophils (Ibrahim *et al.*, 2015) led us to investigate the potential of a2NTD to regulate neutrophil survival.

Our data provide evidence that cancer‐associated a2V prolongs neutrophil survival. Immunohistochemical analysis shows that there is a high expression of a2V that correlates with increased numbers of neutrophils in four different types of cancer, this is particularly prominent in lung cancer. Treatment of neutrophils with recombinant a2NTD enhances the survival of neutrophils by delaying apoptosis. Further analysis to determine the mechanism by which a2NTD is regulating neutrophil apoptosis revealed that a2NTD acts primarily through activation of NF‐κB, main regulator of apoptosis. a2NTD treatment stimulates the production of antiapoptotic proteins, Bcl‐xL and Bcl‐2A1, and prosurvival cytokines, TNF‐α and IL‐8, and downregulates the expression of proapoptotic proteins such as Bax, Apaf‐1, caspase‐3, caspase‐6, and caspase‐7. This retards the mitochondrial apoptosis pathway. We demonstrate for the first time that a cancer‐associated a2V promotes neutrophil survival by regulating the intrinsic apoptosis pathway through activation of NF‐κB.

## 2 Materials and methods

### 2.1 Cells and tissue samples

These studies were approved by the Rosalind Franklin University of Medicine and Science Institutional Review Board. After informed written consent was obtained in accordance with the Declaration of Helsinki, peripheral blood was collected from healthy adult volunteers into sodium heparin vacutainers (Thermo Fisher Scientific Inc., Waltham, MA, USA). Neutrophils were isolated using the dextran/Ficoll technique under endotoxin‐free conditions using Ficoll‐Paque PLUS (Thermo Fisher Scientific) as described previously (Hirota *et al.*, 2005). The neutrophil cell pellet was resuspended in MEM complete media (Invitrogen, Grand Island, NY, USA) containing 10% heat‐inactivated fetal bovine serum (FBS), penicillin (100 U·mL^−1^), streptomycin sulfate (100 mg·mL^−1^), and 2.0 mm glutamine in a cell density of 1 × 10^6^ cell·mL^−1^. Neutrophil purity was 98–99% (CD15/high side scatter) with 99% viability as determined by flow cytometry and trypan blue exclusion. Neutrophils present in serum‐free media were recommended for some experiments. For that purpose, neutrophils were washed two times with MEM serum‐free media and then suspended in the same media. Neutrophils were incubated at 37 °C in a humidified atmosphere containing 5% (v/v) CO_2_.

For immunohistochemistry, paraffin‐embedded human cancer tissue array sections of 5 µm size from four different organs, lung, endometrium, bladder, and kidney, along with two adjacent normal tissues from each organ were procured from BioChain, Newark, CA, USA.

### 2.2 Recombinant a2NTD

a2NTD was expressed and purified from *Escherichia coli* and subjected to endotoxin removal column chromatography (Proteome Resources, Aurora, CO, USA) as previously described (Kwong *et al.*, 2011; Ntrivalas, Derks, *et al.*, 2007a). Limulus assay determined that the endotoxin level is within the reported range of commercially available recombinant proteins. The endotoxin content of a2NTD preparations was 0.0052 ± 0.12 endotoxin units per μg a2NTD or 0.52 ± 0.012 pg·μg^−1^ a2NTD (Kwong *et al.*, 2011). Polymyxin B (binds and inactivates LPS)‐treated a2NTD has the same activity as untreated a2NTD (Kwong *et al.*, 2011). Depletion of a2NTD by immunoadsorption or inactivation of a2NTD by heat demonstrates loss of biological activity (Kwong *et al.*, 2011; Ntrivalas, Derks, *et al.*, 2007a). a2NTD was used in 500 ng·mL^−1^ concentration that was chosen based on a dose–response curve. We found that this concentration gives the highest response based on neutrophils and also based on our previous studies (Ibrahim *et al.*, 2015, 2016; Katara *et al.*, 2014).

The specificity of a2NTD was assessed using bovine serum albumin (BSA) (Fig. S1A,B) or a scramble peptide (Fig. S7) as negative control. Both BSA and the scramble peptide showed no effect on neutrophil survival.

### 2.3 Inhibitors and blocking antibodies

Inhibitors used are listed here as follows: parthenolide, NF‐κB inhibitor (Millipore, Burlington, MA, USA); cycloheximide (Millipore); Z‐IETD‐FMK caspase‐8 inhibitor (BD Biosciences, San Jose, CA, USA); and N‐acetyl‐L‐cysteine, ROS scavenger (Sigma‐Aldrich, St. Louis, MO, USA).

Neutralizing and blocking antibodies are listed here as anti‐human TNF‐α (R&D Systems, Minneapolis, MN, USA), anti‐human IL‐8 (BD Pharmingen, San Jose, CA, USA), anti‐human G‐CSF (Abcam, Cambridge, UK), and anti‐human TLR2 (pattern recognition receptor (PRR)) (Biolegend, San Diego, CA, USA).

### 2.4 Flow cytometry

Fluorescent‐stained cells were analyzed by LSR II FACSscan flow cytometer (BD Bioscience). The cell percentages and the fluorescence intensity measurements were developed with flowjo software (Tree Star, Ashland, OR, USA).

#### 2.4.1 Apoptosis assay

Neutrophil apoptosis was detected by double staining with Pacific Blue Annexin‐V and 7‐AAD (both Biolegend). 0.5 × 10^6^ neutrophils were treated with either PBS (vehicle control) or 500 ng·mL^−1^ a2NTD for eighteen hour at 37 °C in CO_2_ incubator. Pretreatment of neutrophils by inhibitors, and neutralizing or blocking antibodies for 20 min was performed in the required experiments. Cells were suspended in Annexin‐V binding buffer (Biolegend), then stained with Annexin‐V/7‐AAD for 10 min. 250 µL of Annexin‐V binding buffer was added to the cells. Cells were immediately analyzed by LSR II FACSscan flow cytometer (BD Bioscience). The cells were gated into four gates: Annexin‐V/7‐AAD^−/−^ represent live cells, Annexin‐V/7‐AAD^+/−^ represent early apoptotic cells, Annexin‐V/7‐AAD^−/+^ represent late apoptotic cells, and Annexin‐V/7‐AAD^+/+^ represent dead cells. Percentage survival represents the percentage of Annexin‐V/7‐AAD^−/−^ cells.

#### 2.4.2 Intracellular staining

Fixation/Permeabilization Solution Kit (BD Biosciences) was used to fix and permeabilize the cells according to the manufacturer’s recommendation. Briefly, 0.5 × 10^6^ neutrophils were treated with either PBS (vehicle control) or 500 ng·mL^−1^ a2NTD for eighteen hour at 37 °C in CO_2_ incubator. Cells were suspended in 250 µL of fixation/permeabilization solution for 20 min and then washed with Fix/Perm/Wash buffer. Cells were then exposed to direct or indirect staining. Direct immunofluorescence was performed using FITC anti‐human active caspase‐3 (BD Biosciences) for 30 min. The indirect immunofluorescence staining was done in two steps. First, we stain with the primary antibody as anti‐human cleaved caspase‐8 (Asp384) (Cell Signaling, Danvers, MA, USA) or anti‐human cleaved caspase‐9 (Asp315) (Cell Signaling) for 30 min followed by the secondary FITC‐conjugated antibody (Thermo Fisher) for 30 min. Isotype controls showed negative staining.

#### 2.4.3 Surface staining

The expression of Fas and Fas ligand on the surface of neutrophils was performed by direct immunofluorescence using APC anti‐human CD95 (Fas) (Biolegend) or PE anti‐human CD178 (Fas ligand) (eBioscience, Waltham, MA, USA) as described previously (Liles *et al.*, 1996). Briefly, after eighteen‐hour incubation at 37 °C in CO_2_ incubator, 0.5 × 10^6^ of control or a2NTD‐treated neutrophils were washed with ice‐cold PBS. Cells were suspended in ice‐cold staining buffer (Biolegend) and stained with either APC anti‐Fas or PE anti‐Fas ligand for 30 min on ice at dark. After staining, cells were washed with ice‐cold PBS and fixed with 4% paraformaldehyde for 20 min on ice. Isotype controls showed negative staining.

#### 2.4.4 Assessment of mitochondrial membrane potential (MMP)

Mitochondrial membrane potential was measured using the mitochondrial‐specific dual‐fluorescence probe, JC‐1 (Enzo Life Sciences, Farmingdale, NY, USA), based on modified method previously described (Liu *et al.*, 2012). Different dilutions of JC‐1 (ranging from 10 to 1 μm) were used to define the proper JC‐1 concentration that determines specifically the mitochondrial membrane potential. Neutrophils were treated with PBS, a2NTD, or a2NTD/parthenolide for eighteen hour at 37 °C in CO_2_ incubator. 0.25 × 10^6^ neutrophils suspended in 1 mL PBS were incubated with the 2.5 μm JC‐1 for 10 min followed by two washes with PBS. JC‐1 accumulates in mitochondria, selectively generating an orange J‐aggregate emission profile (590 nm per PE channel) in healthy cells. In injured cells, as membrane potential decreases, JC‐1 monomers are generated, resulting in a shift to green emission (529 nm/AF‐488 channel). Aggregate/monomer ratio was calculated (% of PE‐positive cells/% of AF‐488‐positive cells). Freshly isolated neutrophils (> 99% of intact MMP) were used to differentiate the gates between the intact and the disturbed MMP.

#### 2.4.5 Reactive Oxygen Species (ROS) generation

Assay of ROS generation was performed as described previously (Schymeinsky *et al.*, 2007). 1 × 10^6^ neutrophils were pretreated with or without 15 μm N‐acetyl‐l‐cysteine (NAC) (Sigma‐Aldrich). After that, cells were stimulated with PBS (vehicle control) or 500 ng·mL^−1^ a2NTD or FMLP (positive control) one hour at 37 °C in CO_2_ incubator, and incubated with 2.5 μm CM‐H_2_DCFDA (5‐(and‐6)‐chloromethyl‐2′,7′‐dichlorodihydrofluorescein diacetate, acetyl ester) in Hanks' balanced saline solution for 30 min at 37 °C under 5% CO_2_. Data are presented as mean fluorescence intensity (MFI).

### 2.5 Confocal microscopy

Stained cells were imaged by an Olympus Fluoview FV10i Confocal Microscope (Olympus Corporation of the Americas, Center Valley, PA, USA) and analyzed by fv10i fluoview Ver.3.0 software (Olympus Corporation of the Americas).

#### 2.5.1 Assessment of NF‐κB p65 activation

1 × 10^5^ freshly isolated neutrophils suspended in serum‐free MEM were treated with 500 ng·mL^−1^ a2NTD or PBS (vehicle control) with or without the pretreatment by N‐acetyl cysteine (NAC, 15 µm) or by anti‐human TLR2 (10 μg·mL^−1^), and incubated for 30 min or 1 h at 37 °C in CO_2_ incubator. The cells were fixed with 4% paraformaldehyde, cytospun on glass slides, permeabilized by alcohol/acetone, blocked, and stained with anti‐human NF‐κB p65 (Abcam). Alexa Fluor® 488‐conjugated goat anti‐rabbit secondary antibodies (1 : 200 dilution) (Invitrogen, Waltham, MA, USA) were used. The cells were prepared for viewing using ProLong® Gold (Invitrogen) mounting medium containing DAPI.

#### 2.5.2 Assessment of colocalization to mitochondria

1 × 10^5^ isolated neutrophils cultured in complete MEM were treated with 500 ng·mL^−1^ a2NTD or PBS (vehicle control) in the presence or absence of parthenolide (2.5 μm) pretreatment, and incubated for 18 h at 37 °C in CO_2_ incubator. Cells were fixed and processed as mentioned earlier. Cells were then stained with anti‐human TOM20 (mitochondrial marker) (Cell Signaling), anti‐human Bax 6A7 (reacts with Bax of its active conformation; Dumitru *et al.*, 2012; Santa Cruz Biotechnology, Dallas, TX, USA), or anti‐human cytochrome *c* (Biolegend). Alexa Fluor® 488‐conjugated donkey anti‐mouse and Alexa Fluor® 594‐conjugated goat anti‐rabbit secondary antibodies (1 : 200 dilution) (Invitrogen) were used. The cells were prepared for viewing using ProLong® Gold (Invitrogen) mounting medium containing DAPI.

### 2.6 Fluorescence microscopy

1 × 10^6^ freshly isolated neutrophils cultured in complete MEM were treated with 500 ng·mL^−1^ a2NTD or PBS (vehicle control) in the presence or absence of parthenolide pretreatment (2.5 μm). After four‐hour incubation at 37 °C in CO_2_ incubator, cells were fixed, cytospun on glass slide, and processed as mentioned earlier. Cells were directly stained with FITC anti‐human Bcl‐xL (Abcam) or with anti‐human c‐FLIP (Enzo Life Sciences) followed by secondary Alexa Fluor® 488‐conjugated donkey anti‐mouse (1 : 200 dilution) (Invitrogen). Cells that were incubated for eighteen hour at 37 °C in CO_2_ incubator were stained by anti‐human cleaved poly (ADP‐ribose) polymerase (PARP) (Asp 214) (BD Pharmingen), anti‐human Bax 6A7 (Santa Cruz Biotechnology), or anti‐human cleaved caspase‐9 (Asp315) (Cell Signaling) followed by staining with secondary antibodies (1 : 200 dilution) Alexa Fluor® 488‐conjugated donkey anti‐mouse or Alexa Fluor® 594‐conjugated goat anti‐rabbit (Invitrogen). Stained cells were imaged on Olympus microscope and analyzed using nis‐elements software (Nikon Inc, Melville, NY, USA).

### 2.7 Immunohistochemistry of human cancer tissues

Immunohistochemical staining of paraffin‐embedded human tissue sections of 5 µm size was performed using Dako EnVision™+ Dual Link System‐HRP (Dako, Carpinteria, CA, USA) system according to the manufacturer’s protocol. Anti‐a2V (antibody specific for amino acids 142–344, the N‐terminal region of the a2 isoform of the ‘V0a’ subunit; a2NTD) was generated as previously described (Ntrivalas, Gilman‐Sachs, Kwak‐Kim, and Beaman, 2007b), and anti‐human neutrophil elastase NP‐57 (Santa Cruz Biotechnology). Before the primary antibody incubation, heat‐induced epitope retrieval in sodium citrate buffer pH = 6 was performed (for a2NTD detection, see Jaiswal *et al.*, 2014). Tissue sections stained with isotype control antibodies (R&D Systems) at the same concentration as the primary antibodies showed negative staining. Light photomicroscope Leica ICC50 W (Leica Microsystems Inc., Buffalo Grove, IL, USA) with a built‐in high‐resolution camera was used to evaluate the immunostaining. Scale bars were calculated using leica las ez software (Leica Microsystems Inc.).

Quantification of neutrophil count was determined by counting positively stained cells from at least five different 200× fields per section manually and confirmed by using fiji software (Schindelin *et al.*, 2012). Data are shown as means ± SEM. Quantification of the intensity of the staining of a2NTD expression was performed as described previously (Nguyen *et al.*, 2013; Varghese *et al.*, 2014). Briefly, the intensity of the staining in the stained areas was measured by the color deconvolution algorithm using fiji software and reciprocal intensity (R.I.) was calculated by subtracting the chromagen intensity (*y*) from the maximum intensity, R.I. = 255 – *y*. R.I. is positively correlated with the intensity of the staining (Nguyen *et al.*, 2013). The analysis was performed in at least ten different 400× fields per each section.

### 2.8 RNA isolation and real‐time PCR

After treating neutrophils with PBS (control) or recombinant a2NTD for 4‐h incubation at 37 °C in 5% CO2 incubator, total RNA was isolated using RNeasy Mini Kit (Qiagen, Germantown, MD, USA) and single‐stranded cDNA was synthesized using QuantiTect Reverse Transcription Kit (Qiagen) according to the manufacturer’s instruction. Quantitative real‐time PCR (qRT‐PCR) was performed, using Universal Fast PCR Master Mix Reagent (Applied Biosystems, Waltham, MA, USA) and cDNA‐specific FAM‐MGB‐labeled TaqMan primer sets (Applied Biosystems) for different genes, and VIC‐MGB‐labeled *18s rRNA* was used as endogenous control. In some experiments, parthenolide (2.5 μm) or anti‐TLR2 (10 μg·mL^−1^) was used for pretreatment of the cells. The data were analyzed using the comparative CT (ΔΔ*C*_t_) method using *18s rRNA* as the endogenous control.

### 2.9 Cytokine/growth factor bioassay

The secretion of TNF‐α and IL‐8 was analyzed by Human ELISA MAX™ Deluxe Kit (Biolegend) in the supernatant of neutrophils (1 × 10^6^ cell·mL^−1^) collected after designed time incubation at 37 °C in a humidified atmosphere containing 5% (v/v) CO_2_. The assay was performed on ELISA plate reader according to the manufacturer’s instructions. Equal volumes from cell supernatant were used for the assay.

The secretion of G‐CSF and GM‐CSF was analyzed by Milliplex MAP Kit (Millipore) in the supernatant of neutrophils (1 x 10^6^ cell·mL−1) collected after four hour or overnight incubation at 37 °C in a humidified atmosphere containing 5% (v/v) CO_2_. Then, the supernatants were assayed on a MAGPIX instrument (Millipore) as per the instructions provided by the manufacturer. Equal volumes from cell supernatant were used for the assay.

### 2.10 Quantification of NF‐κB p65 (pSer 536)

Quantification of NF‐κB p65 phosphorylation at Ser 536 in neutrophil lysates was performed by NF‐κB p65 (Total/Phospho) Human InstantOne™ ELISA Kit (Thermo Fisher Scientific). Neutrophil lysates were collected from 2 × 10^6^ cells after indicated time point of treatment by a2NTD (500 ng·mL^−1^) or PBS (control) in the presence or absence of 15 μm NAC. Protein concentration of neutrophil lysates was estimated using Pierce™ BCA Protein Assay Kit (Thermo Fisher Scientific), and equal amounts of protein were used for quantification of NF‐κB p65 (pSer 536) as per the manufacturer’s instructions. The assay was performed on ELISA plate reader according to the manufacturer’s instructions.

### 2.11 Immunoblotting

Equal amounts of total protein lysates were mixed with sample buffer, heated at 95 °C for 5 min, and loaded to SDS/PAGE and transferred to nitrocellulose. The membranes were blocked in protein‐free PBS blocking buffer (Thermo Scientific, Rockford, IL, USA) for 1 h at room temperature. Primary antibodies were incubated in the blocking buffer for 16 h at 4 °C. Secondary antibodies were incubated in the same blocking buffer for 1 h at room temperature. Protein signals were detected using an Odyssey imaging instrument, and the intensities of the immunoreactive bands were quantified by densitometric analysis using instrument software (Li‐COR Biosciences, Lincoln, NE, USA).

### 2.12 TIMER analysis

Tumor IMmune Estimation Resource (TIMER, available at http://cistrome.org/TIMER) (Finotello and Trajanoski, 2018; Li *et al.*, 2016; Li *et al.*, 2017) was used to investigate the correlation between the a2V gene expression and neutrophil infiltration in 515 RNA‐seq human lung adenocarcinoma (LUAD) samples from The Cancer Genome Atlas (TCGA). The scatterplot shows the purity‐corrected partial Spearman’s correlation and statistical significance that is provided within the data sets in TIMER.

### 2.13 Statistical analysis

Statistical analysis was performed with two‐tailed paired or unpaired Student’s *t*‐test or Mann–Whitney test. Error bars show means ± SEM. *P* value <0.05 was considered significantly different. Correlation analysis was performed by linear regression analysis. The chi‐square statistical analysis was performed to distinguish significant difference between the differentiation state trends between high and low neutrophil count groups. Statistical tests were performed using graphpad prism 5 (GraphPad Software, Inc., San Diego, CA, USA).

## 3 Results

### 3.1 a2V expression and the presence of TAN in different human cancer types

**Figure 1:**
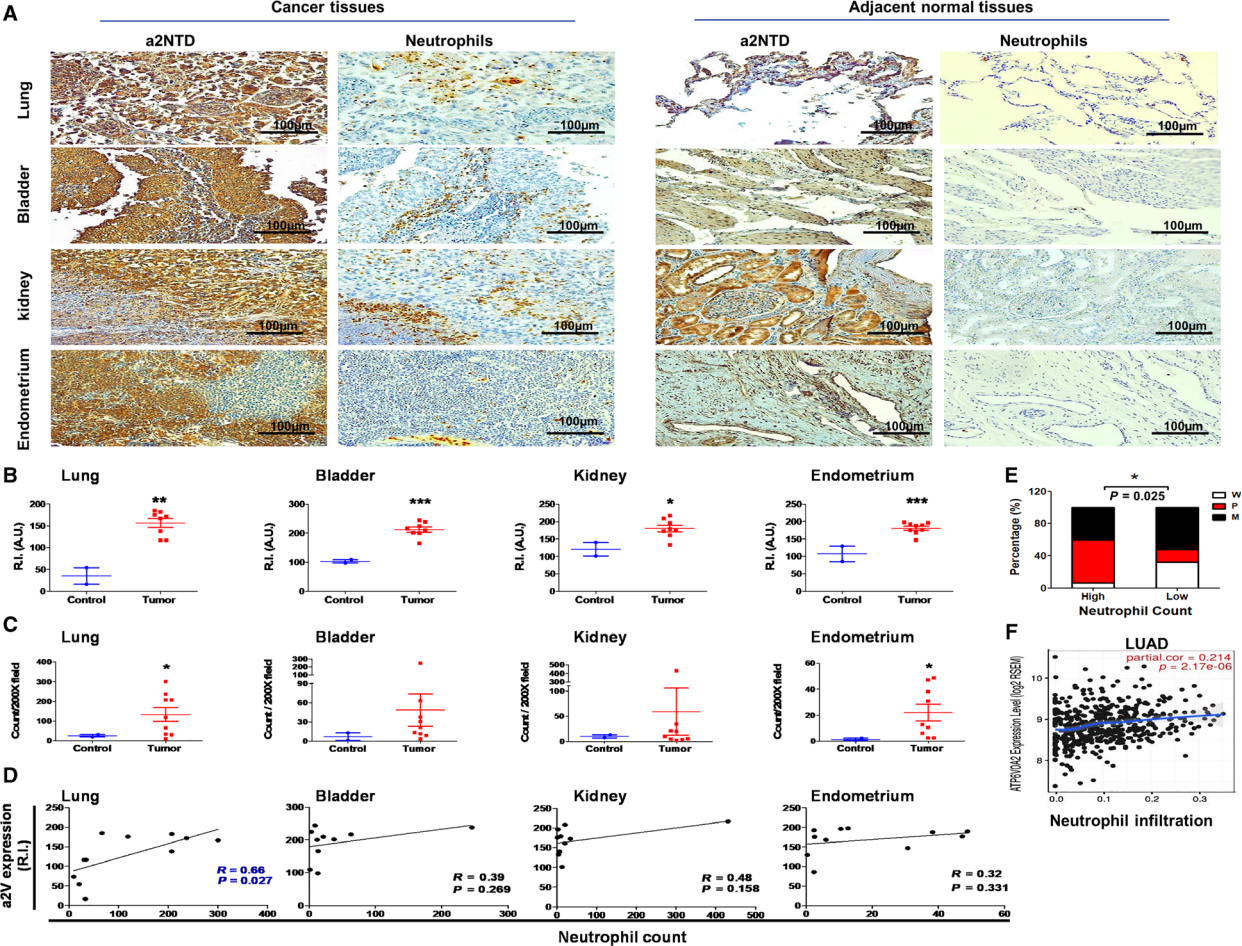
a2V expression and the presence of TAN in different human cancer types. (A) Immunohistochemical staining of a2V and neutrophil using specific antibodies toward the N‐terminal domain of a2V and neutrophil elastase in paraffin‐embedded tissue array sections of different human cancer types was examined microscopically. Original magnification 200×; scale bars: 100 μm. Brown represents positive staining. (B) a2V expression was quantified by measuring the mean intensity of the stained areas from at least ten different 400× fields per section using fiji software. Results represented as reciprocal intensity (R.I.) ± SEM. Statistical significance of group differences assessed by two‐tailed unpaired Student’s *t*‐test, *P < 0.05, **P < 0.01, and ***P < 0.001, as compared to the adjacent normal tissues. (C) Quantification of neutrophils was determined by counting positively stained cells from at least five different 200× fields per section. Data are shown as means ± SEM. Statistical significance of group differences assessed by two‐tailed unpaired Student’s *t*‐test with Welch’s correction, *P < 0.05, as compared to the adjacent normal tissues. Tumor tissues *n* = 35 and control tissues *n* = 9. (D) The correlation between the a2V expression and the neutrophil count in each tissue is determined by linear regression analysis. (E) The relation between neutrophil count and differential state of the tumors. High and low represent ≥ 35 and < 35 mean neutrophil count, respectively. Differentiation state represented as W, well; M, moderate; and P, poor. The chi‐square statistical analysis was performed, *P < 0.05. (F) Purity‐corrected partial Spearman’s correlation between the a2V expression in lung adenocarcinoma (LUAD) and neutrophil infiltration. rsem is a software package used for accurate transcript quantification from RNA‐seq data.

Identifying the tumor‐associated proteins that regulate tumor‐associated neutrophils (TAN) is important to precisely target this distinct population of cells. First, we wanted to investigate the intensity of the expression of the a2 isoform of vacuolar ATPase (a2V) and the presence of neutrophils in human cancer. Also, we wanted to address whether there is a relation between a2V expression and neutrophil count in cancer tissues. We performed immunohistochemical analysis of a2V and neutrophils using a specific antibody toward the N‐terminal domain of a2V and neutrophil elastase, specific marker for neutrophils. We performed this analysis in cancer tissue arrays collected from four different organs, lung, bladder, kidney, and endometrium (Fig. 1A). Quantification of the intensity of a2V expression was significantly higher in tumor tissues in all four examined organs compared with the a2V expression in the control tissues (Fig. 1B). Importantly, we found a similar pattern of increased neutrophil count in tumor tissues relative to control (Fig. 1C). In addition, comparing the neutrophil count with the differentiation state of the tumor cells revealed that the tissues that are heavily infiltrated by neutrophils (≥ 35 mean count) are either poorly (53.3%) or moderately (40%) differentiated that indicates highly aggressive tumors (Fig. 1E). One tissue was highly infiltrated with neutrophils but was classified as well‐differentiated tumor, and that was a bladder cancer tissue. Furthermore, we performed a correlation analysis between the expression of a2V and the neutrophil count in each cancer type (Fig. 1D). This analysis revealed a general positive correlation between a2V expression and the neutrophil count in all types of tested cancer. This correlation was statistically significant in lung cancer with a correlation coefficient of 0.66. We validated these results by performing correlation analysis between the a2V gene expression and neutrophil infiltration in human lung adenocarcinoma samples from TCGA using timer software (Fig. 1F). The results showed a significant positive correlation between a2V expression and neutrophil infiltration. Together, these data demonstrate the positive relationship between cancer‐associated a2V expression and the TAN presence in different types of cancer.

### 3.2 a2NTD prolongs neutrophil survival

**Figure 2:**
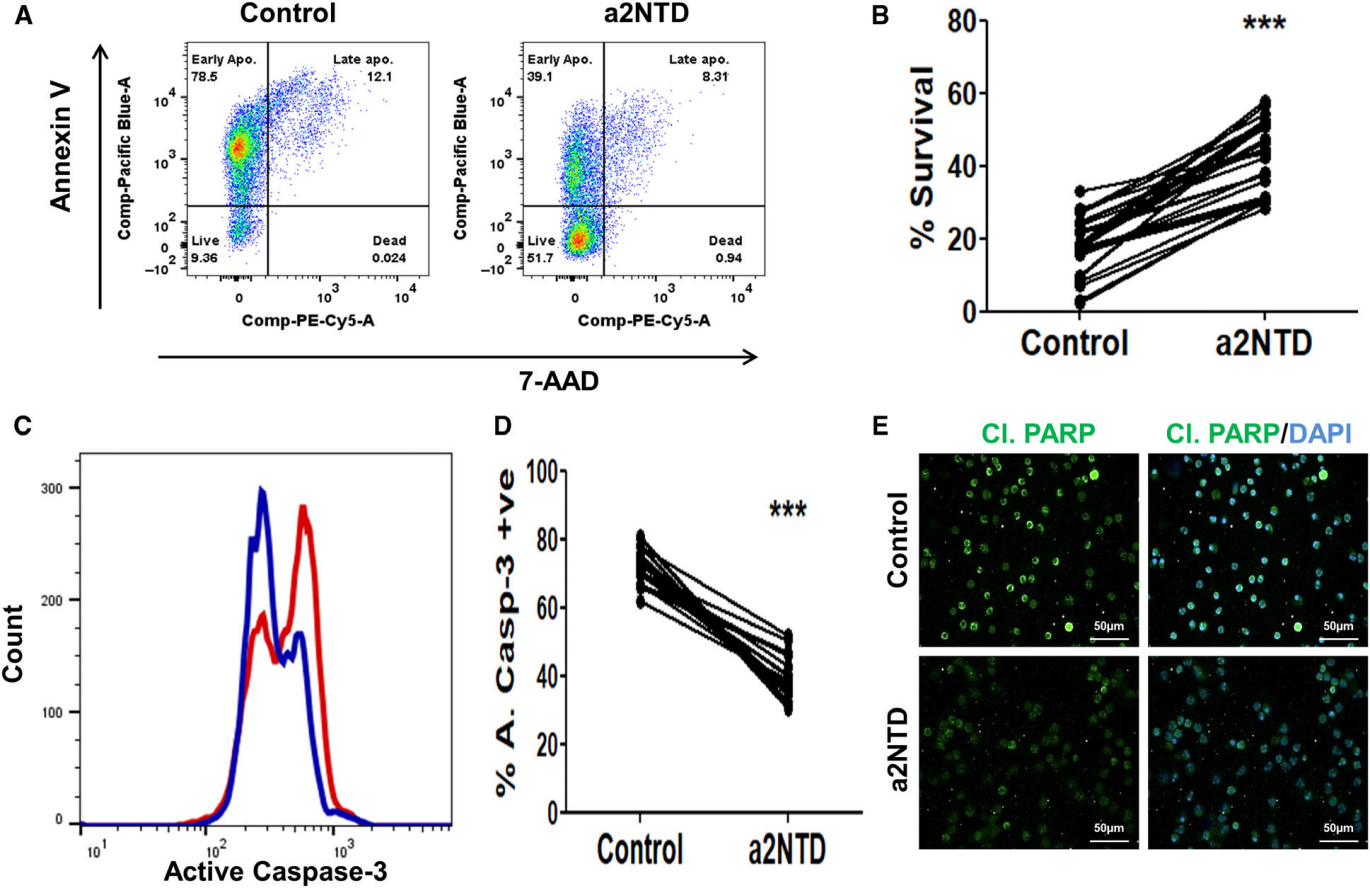
a2NTD prolongs neutrophil survival. (A) Representative dot plots of flow cytometry analysis to assess neutrophil apoptosis. Isolated neutrophils were treated with PBS (control) or a2NTD. Annexin‐V/7‐AAD staining was used to identify viable cells (Annexin‐V/7‐AAD^−/−^), early apoptotic cells (Annexin‐V/7‐AAD^+/−^), late apoptotic cells (Annexin‐V/7‐AAD^+/+^), and dead cells (Annexin‐V/7‐AAD^−/+^). Experiments were done in duplicate (*n* = 14). (B) Quantification of the percentage survival of Annexin‐V^−^/7‐AAD^−^ cells of neutrophils. Statistical significance of group differences determined by two‐tailed paired Student’s *t*‐test, ***P < 0.001 as compared to control neutrophils (*n* = 14). (C) Representative flow cytometry analysis of active caspase‐3 expressed by a2NTD‐treated neutrophils (blue) in comparison with control (red). (D) Quantitative analysis of the percentage of the active caspase‐3‐positive cells. Experiments were done in duplicate (*n* = 12). Statistical significance of group differences determined by two‐tailed paired Student’s *t*‐test, ***P < 0.001 as compared to control neutrophils. (E) Immunofluorescence analysis of cleaved PARP using fluorescence microscope (*n* = 3). Original magnification 400×; scale bars: 50 μm, cleaved PARP (green); nucleus (DAPI, blue). 500 ng·mL^−1^, 18‐h incubation of a2NTD was used in neutrophil treatment, unless otherwise stated.

Neutrophils are short‐living immune cells, but they acquire a long life span in cancer sites (Fridlender and Albelda, 2012; Pylaeva *et al.*, 2016; Zhang *et al.*, 2016). Our demonstration that a high level of a2V expression in tumor tissues was associated with an increased neutrophil population in different types of cancer raised the question whether this relation is associated with increased neutrophil survival in tumor site. Treatment of neutrophils isolated from healthy donors with a recombinant a2NTD delayed neutrophil spontaneous apoptosis and enhanced the neutrophil survival by 1.3‐ to 13.2‐fold (Fig. 2A,B and Fig. S1A,B). This enhancement in neutrophil survival was dependent on a2NTD concentrations (Fig. S1C). We confirmed the effect of a2NTD on neutrophil survival by measuring the percentage of neutrophils that express intracellular active caspase‐3 by flow cytometry (Fig. 2C,D). We found a significant decrease in the percentage of neutrophils expressing active caspase‐3 upon a2NTD treatment compared with the control neutrophils (38.5% ± 1.35 and 73% ± 0.99, respectively). Also, we evaluated the time course of a2NTD treatment along with a dose–response approach. We assessed the viability of neutrophils and the active caspase‐3 expression in at least 5 different time points (0, 2, 5, 18, 24, 48 h) after treatment with different concentrations of a2NTD (0, 100, 500, 2500 ng·mL^−1^). Our results showed that control neutrophils started to undergo apoptosis after 5‐h incubation; on the other hand, a2NTD maintained neutrophil survival and inhibited its spontaneous apoptosis at this time point. At the later time points, a2NTD delayed neutrophil apoptosis significantly and this effect was extended to 48‐h treatment incubation compared with the control neutrophil. The best response toward a2NTD was prominent at 18‐h incubation and at 500 ng·mL^−1^ concentration. The 100 ng·mL^−1^ concentration of a2NTD showed lesser effect on maintaining neutrophil survival compared with higher concentrations, and there was no significant difference between the effect of 500 or 2500 ng·mL^−1^ of a2NTD on neutrophil survival, and we had consistent results when we assessed the percentage of active caspase‐3 (Fig. S2). Further, we evaluated the levels of cleaved poly (ADP‐ribose) polymerase (Cl. PARP), one of the main cleavage targets of caspase‐3 (Nicholson *et al.*, 1995; Tewari *et al.*, 1995) by immunofluorescence microscopy. a2NTD treatment protects PARP from cleavage compared with control neutrophils (Fig. 2E). Together, these data confirm the prosurvival effect of a2NTD on neutrophils.

### 3.3 a2NTD maintains neutrophil survival by the activation of NF‐κB

**Figure 3:**
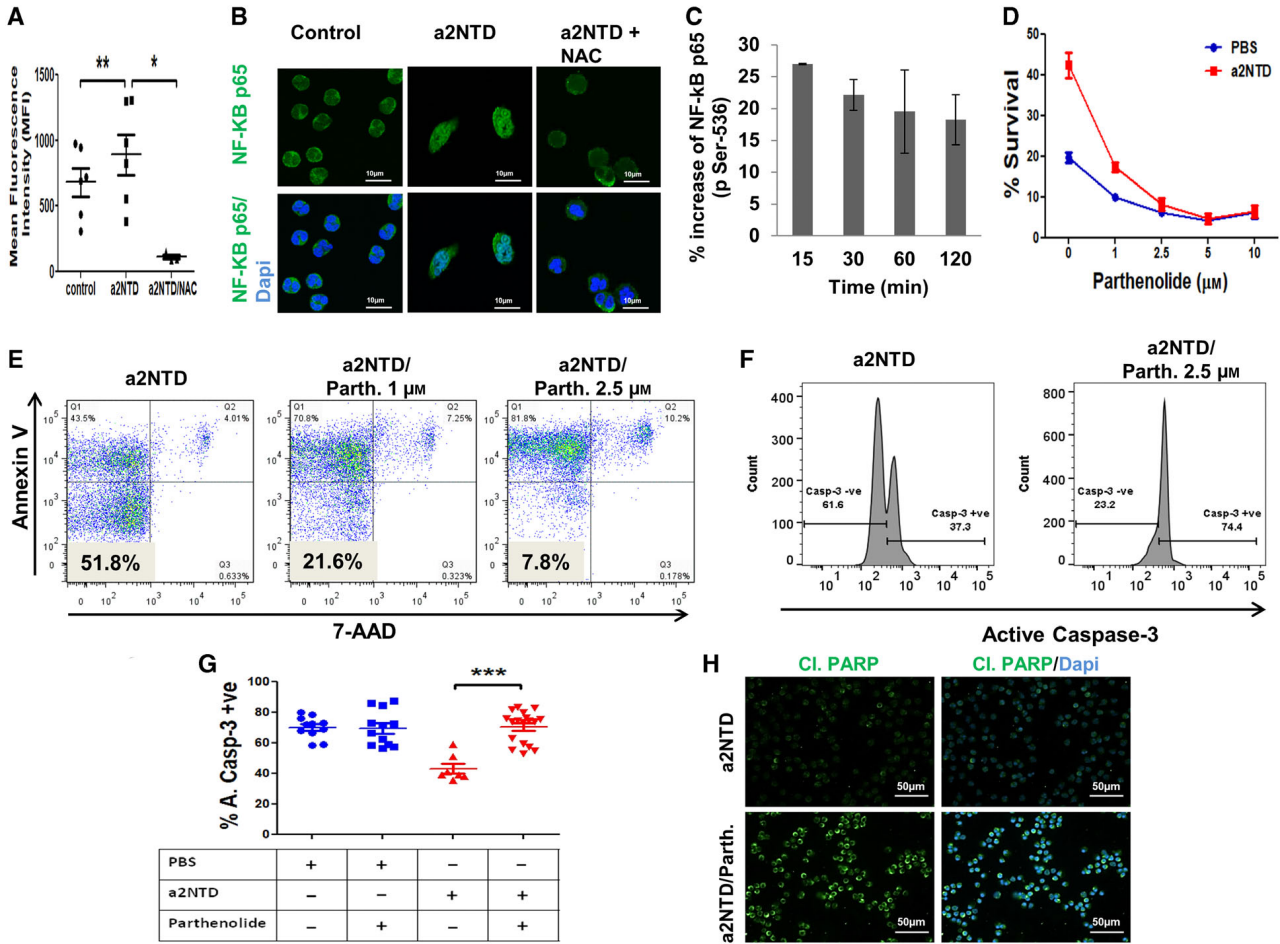
a2NTD maintains neutrophil survival by the activation of NF‐κB. (A) Flow cytometry analysis of ROS generation by control or a2NTD‐treated neutrophils with or without 15 μm NAC pretreatment using CM‐H_2_DCFDA. Results from at least three independent experiments were done in duplicate and presented as the mean fluorescence intensity. Statistical significance of group differences determined by two‐tailed paired Student’s *t*‐test, *P < 0.05, **P < 0.01. (B) Immunofluorescence analysis of NF‐κB p65 expression was performed after 1‐h treatment of neutrophils with a2NTD (500 ng·mL^−1^) or PBS (control) in the presence or absence of N‐acetyl cysteine (NAC, 15 μm). NF‐κB p65 (green), nucleus (DAPI, blue), and colocalization of NF‐κB p65 with the nucleus represents NF‐κB p65 activation. A representative image is presented from 3 different experiments, scale bars: 10 μm. (C) Quantification of NF‐κB p65 phosphorylation at Ser 536 in neutrophil lysates by ELISA after different time points of stimulation by a2NTD (500 ng·mL^−1^) or PBS (control). Data were collected from at least 3 independent experiments and are reported as the mean of percentage increase in NF‐κB p65 (pSer 536) relative to control ± SEM. (D) Percentage survival of neutrophils treated with a2NTD (500 ng·mL^−1^, 18 h) or PBS (control) after the inhibition of NF‐κB by different concentrations of parthenolide (*n* = 3), data presented as mean ± SEM. (E) Representative dot plots show the effect of the inhibition of NF‐κB on the survival of the a2NTD‐treated neutrophils (*n* = 3). (F) Representative histograms show the active caspase‐3 expression in a2NTD‐treated neutrophils in the presence or absence of parthenolide (2.5 μm). (G) Percentage of active caspase‐3‐positive neutrophils (apoptotic cells) after treatment with a2NTD in the presence or absence of parthenolide (*n* = at least 7). Data plotted as mean ± SEM. Statistical significance of group differences determined by two‐tailed paired Student’s *t*‐test, ***P < 0.001 as compared to control neutrophils. (H) Immunofluorescence staining of cleaved PARP using fluorescence microscope (*n* = 3). Original magnification 400×; scale bars: 50 μm, cleaved PARP (green); nucleus (DAPI, Blue). 500 ng·mL^−1^, 18‐h incubation of a2NTD was used in neutrophil treatment, unless otherwise stated. Neutrophils were treated by parthenolide or NAC for 15 min prior the stimulation with a2NTD.

NF‐κB is a critical regulator of apoptosis in neutrophils, and we have shown that a2NTD activates NF‐κB (Cowburn *et al.*, 2004; Ibrahim *et al.*, 2016; McCracken and Allen, 2014; Ward *et al.*, 1999). We therefore investigated the possibility that a2NTD can improve neutrophil survival via NF‐κB activation. Activation of NF‐κB can be derived by reactive oxygen species (ROS) generation (Morgan and Liu, 2011). First, we investigated the effect of a2NTD on ROS generation by a quantitative flow cytometry assay. Treatment with a2NTD generates increased levels of ROS in neutrophils compared with the control, and pretreatment of the cells with N‐acetyl cysteine (NAC), ROS scavenger, inhibited the a2NTD effect on ROS generation (Fig. 3A, Fig. S3A). Second, immunofluorescence analysis revealed that NAC negated the activation of NF‐κB p65 (translocation to the nucleus) upon a2NTD treatment (Fig. 3B). Furthermore, using a quantitative ELISA we demonstrated that a2NTD treatment led to activation of NF‐κB p65 by phosphorylation (P‐p65) at ser‐536 by ~ 27% increase. This effect was rapid, with increased levels of P‐p65 within 15 min, and sustained to 2 h (Fig. 3C). On the other hand, there was no change in the levels of P‐p65 in cells treated with NAC relative to control (Fig. S3B). In order to confirm that this NF‐κB activation is involved in the prosurvival effect of a2NTD on neutrophils, we pretreated the cells with different concentrations of parthenolide, specific NF‐κB inhibitor prior to a2NTD treatment. We assessed the survival rate of neutrophils using Annexin‐V/7‐AAD and quantified the active caspase‐3 using flow cytometry assays (Fig. 3D–G). Parthenolide completely inhibited the prosurvival effect of a2NTD on neutrophils. Also, parthenolide treatment restored the cleavage of PARP as the control (Fig. 3H). Moreover, neutrophil treatment with cycloheximide (protein translation inhibitor) decreased the percentage survival of a2NTD‐treated neutrophils (Fig. S3C). That suggests that a2NTD treatment stimulates the synthesis of prosurvival proteins by neutrophils. Altogether, these results showed that a2NTD activates NF‐κB in neutrophils by ROS generation and that activation was involved in the prosurvival effect of a2NTD on neutrophils.

### 3.4 a2NTD treatment regulates the expression of anti‐ and proapoptotic proteins in neutrophils

**Figure 4:**
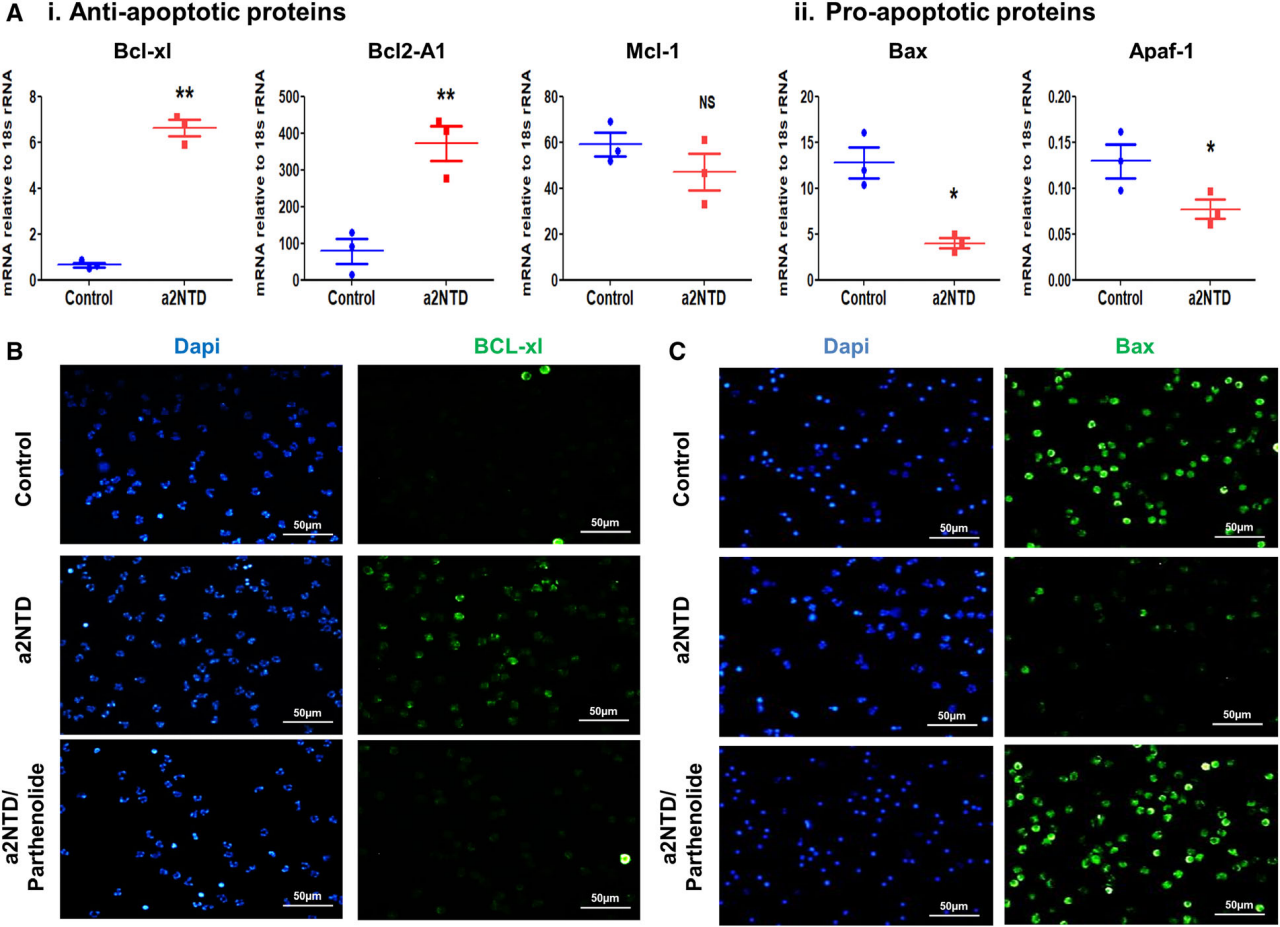
a2NTD treatment regulates the expression of proteins regulating the intrinsic pathway of apoptosis in neutrophils. Quantitative real‐time PCR was performed to assess the mRNA gene expression of (A.i) antiapoptotic proteins, Bcl2‐A1, Bcl‐xL, and MCL‐1; and (A.ii) proapoptotic proteins, Bax and Apaf‐1, in a2NTD or PBS (control)‐treated neutrophils for 4 h. Data reported as mean of mRNA expression relative to 18s rRNA ± SEM from at least three different independent experiments, each was done in triplicate. Statistical significance of group differences determined by two‐tailed Mann–Whitney test, *P < 0.05, **P < 0.01 compared to the control neutrophils. (B, C) Immunofluorescence analysis of Bcl‐xl and BAX, after 4‐ and 18‐h incubation, respectively, in a2NTD‐treated neutrophils with or without 2.5 μm parthenolide. Bcl‐xl and BAX are green, nucleus (DAPI, blue). Imaging was done by fluorescence microscope, original magnification 400×, scale bars: 50 μm (*n* = 3).

The balance between the anti‐ and proapoptotic proteins is an important factor that decides the fate of the cells (Geering and Simon, 2011). NF‐κB pathway regulates the expression of the Bcl‐2 family proteins, important regulators of apoptosis (Chen *et al.*, 2000; Cowburn *et al.*, 2011; Hoesel and Schmid, 2013). Therefore, we checked the mRNA expression of different anti‐ and proapoptotic proteins by quantitative RT‐PCR (Fig. 4A). a2NTD treatment upregulated the gene expression of Bcl‐xL and Bcl2‐A1 by 10.4‐ and 9.1‐fold, respectively, compared with control neutrophils. On the other hand, Mcl‐1 expression did not change. The expression of Bax and Apaf‐1, proapoptotic proteins, was significantly decreased upon a2NTD treatment in comparison with control (3.3‐ and 1.7‐fold decrease, respectively). Also, we demonstrated that the expression of different caspases decreased in neutrophils after a2NTD treatment. Caspase‐3, caspase‐6, and caspase‐7 mRNA expression decreased significantly in a2NTD‐treated cells by 1.6‐, 1.5‐, and 4‐fold, respectively (Fig. S1D). In order to investigate the role of NF‐κB on regulating the expression of these proteins in a2NTD‐treated neutrophils, we pretreated the cells with parthenolide. The mRNA expression of the antiapoptotic proteins such as Bcl‐xL and Bcl2‐A1 decreased upon parthenolide treatment (Fig. S4A,B). Consistently, Bax, caspase‐8, and caspase‐3, proapoptotic proteins, mRNA expressions were increased after inhibition of NF‐κB (Fig. S4C–E). With regard to control cells, NF‐κB inhibition did not affect the expression of these proteins. Furthermore, immunofluorescence staining of Bcl‐xL and Bax showed consistent results with the mRNA data (Fig. 4B,C). a2NTD‐treated neutrophils (a2NuΦ) showed high expression of Bcl‐xL after four hours of treatment and low expression of Bax after eighteen hours of treatment, and this effect was reversed upon NF‐κB inhibition. We confirmed these data by western blot analysis of caspase‐3, Bax, and cleaved PARP (Fig. S4F), and we found consistent results with our mRNA data and the immunofluorescence analysis. Together, these data demonstrate that a2NTD treatment reprogrammed the neutrophil transcription of anti‐ and proapoptotic proteins in a way that supports neutrophil survival.

### 3.5 a2NTD treatment retards the mitochondrial pathway of apoptosis in neutrophils

**Figure 5:**
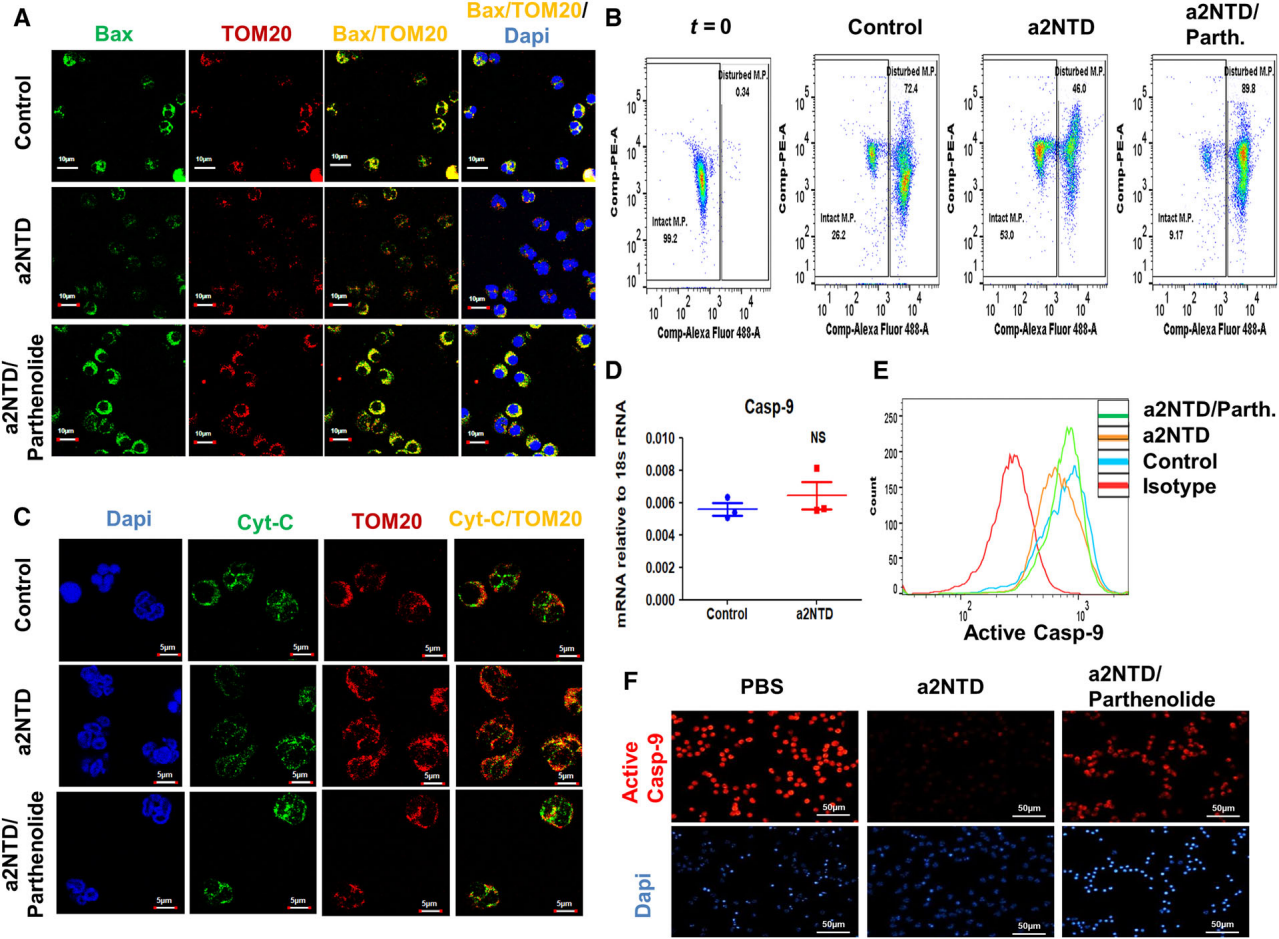
a2NTD treatment retards the mitochondrial pathway of apoptosis in neutrophils. (A) Immunofluorescence analysis of Bax and Tom20 (translocase of the outer membrane, specific marker for the mitochondria) in neutrophils after a2NTD treatment with or without parthenolide (2.5 μm) for 18 h. Colocalization of Bax with Tom20 (yellow) indicates translocation of Bax from cytoplasm to mitochondria. Representative images taken by confocal microscope, scale bars: 10 μm (*n* = 3). (B) Flow cytometry assessment of mitochondrial membrane potential in neutrophils by the fluorescence probe JC‐1 at before (*t* = 0) or after 18‐h treatment with PBS (control), a2NTD, or a2NTD with parthenolide. Representative dot plots of 2 individual experiments done in duplicates present the JC‐1 aggregates (PE^Hi^) on *y*‐axis and JC‐1 monomers (AF‐488^Lo^) on *x*‐axis. (C) Immunofluorescence staining of cyt‐*C* (green) and Tom20 (red) in neutrophils after a2NTD treatment with or without parthenolide (2.5 μm) for 18 h. The release of cyt‐*C* is determined by the free green fluorescence (not colocalized with red fluorescence). Representative images taken by confocal microscope, scale bars: 5 μm (*n* = 3). (D) Assessment of the mRNA expression of Caspase‐9 by qRT‐PCR after 4‐h treatment of a2NTD. Results presented as mean of mRNA expression relative to 18s rRNA ± SEM (*n* = 3). No statistical significance of group differences determined by two‐tailed Mann–Whitney test. (E, F) Evaluation of active caspase‐9 expression in neutrophils after a2NTD treatment with or without parthenolide by flow cytometry or immunofluorescence microscopy with scale bars: 50 μm, respectively (*n* = 3).

The intrinsic apoptosis pathway is tightly regulated by Bcl2 family proteins that regulate the mitochondrial membrane integrity (Cowburn *et al.*, 2011). Since we showed that a2NTD treatment regulates the expression of some Bcl2 family members in neutrophils, we further investigated the effect of a2NTD on the mitochondrial pathway of apoptosis. In this pathway, Bax that resides in the cytoplasm translocates to the mitochondrial membrane. This translocation to the mitochondrial membrane disrupts the mitochondrial membrane potential (Dewson and Kluck, 2009). Immunofluorescence analysis demonstrated that Bax was highly colocalized with the mitochondria in the control neutrophils and in parthenolide‐treated a2NuΦ (Fig. 5A). In contrast, a2NTD treatment significantly decreased Bax translocation to the mitochondria. The assessment of the mitochondrial membrane potential by flow cytometry using JC‐1 dye showed that a2NTD treatment increased the percentage of cells with high membrane potential by ~ 2‐fold and that it was dependent on NF‐κB activation (Fig. 5B and Fig. S6A,B). Disruption in the mitochondrial membrane potential leads to the leakage of some apoptotic driver proteins including cytochrome *c* (cyt‐*c*) from mitochondria. Cyt‐*c* is important for the apoptosome assembly and the activation of caspase‐9 (Dewson and Kluck, 2009). We assessed the levels of cytosolic cyt‐*c* by immunofluorescence staining (Fig. 5C). Cyt‐*c* was mainly sequestered in the mitochondria in a2NuΦ compared with the increased levels of cytoplasmic cyt‐*c* in the control neutrophils and upon NF‐κB inhibition in a2NuΦ. Furthermore, qRT‐PCR demonstrated that there was no significant difference in caspase‐9 gene expression upon a2NTD treatment of neutrophils (Fig. 5D). Flow cytometry analysis and immunofluorescence staining revealed that a2NTD treatment decreases the levels of active caspase‐9 in neutrophils and this phenotype was reversed by NF‐κB inhibition (Fig. 5E,F). Collectively, these data demonstrate that a2NTD treatment decreased the intrinsic pathway of apoptosis in neutrophils and that it was dependent on NF‐κB activation.

### 3.6 Autocrine stimulation of TNF‐α and IL‐8 participates in the maintenance of survival of a2NTD‐treated neutrophils

**Figure 6:**
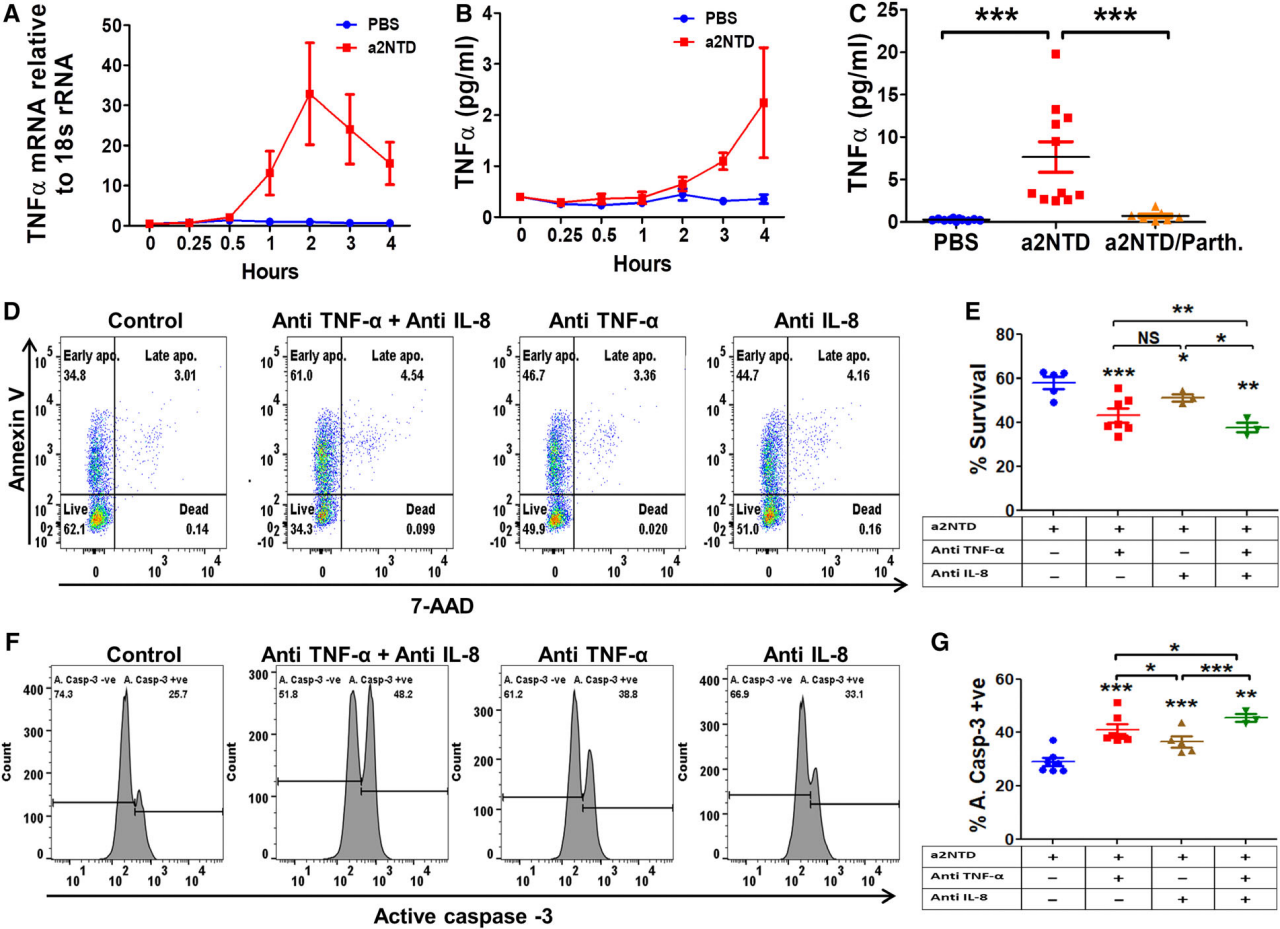
Autocrine stimulation of TNF‐α and IL‐8 participates in the maintenance of survival of a2NTD‐treated neutrophils. Quantification of TNF‐α (A) gene expression by qRT‐PCR and (B) protein levels (pg·mL^−1^) by ELISA in neutrophils after different stimulation periods by a2NTD. Data presented as mean ± SEM (*n* = 3). Statistical significance of group differences determined by two‐tailed Mann–Whitney test (gene expression assays) or two‐tailed paired Student’s *t*‐test (protein level assays). (C) TNF‐α secretion by neutrophils after treatment with a2NTD (500 ng·mL^−1^, 18 h) in the presence or absence of parthenolide (2.5 μm). Results presented as mean ± SEM (*n* = at least 7). (D) Apoptosis assessment of a2NTD‐treated neutrophils by Annexin‐V/7‐AAD after treatment with neutralizing antibodies toward TNF‐α (5 μg·mL^−1^) and/or IL‐8 (20 μg mL^−1^). (E) Quantification of the percentage survival of neutrophils from the Annexin‐V/7‐AAD flow cytometry experiment. Results are shown as mean ± SEM (*n* = at least 3). Statistical significance of group differences determined by two‐tailed paired Student’s *t*‐test. (F) Active caspase‐3 expression in a2NTD‐treated neutrophils after adding TNF‐α (5 μg·mL^−1^) and/or IL‐8 (20 μg·mL^−1^) by flow cytometry. (G) Quantification of the percentage of active caspase‐3‐positive neutrophils (apoptotic cells). Results are shown as mean ± SEM (*n* = at least 3). Statistical significance of group differences determined by two‐tailed paired Student’s *t*‐test, *P < 0.05, **P < 0.01, ***P < 0.001.

TNF‐α and IL‐8 act as a prosurvival cytokines for neutrophils (Elbim and Estaquier, 2010). We have shown previously that IL‐8 expression and secretion were induced in a2NuΦ (Ibrahim *et al.*, 2016). We examined the kinetics of the expression and the secretion of TNF‐α by qRT‐PCR and ELISA, respectively (Fig. 6A,B). We found that TNF‐α mRNA expression started to be detected in neutrophils after one hour of a2NTD treatment. A significant increase in the secreted levels of TNF‐α started after three hours of a2NTD treatment and reached 27.3‐fold increase after overnight culture. This increase in the secretion of TNF‐α was negated upon NF‐κB inhibition (Fig. 6C). In order to test whether TNF‐α and IL‐8 participate in supporting neutrophil survival of a2NuΦ, we pretreated the cells with IL‐8‐ and/or TNF‐α‐neutralizing antibodies (Fig. 6D,E). Upon assessment of neutrophil survival by Annexin‐V/7‐AAD flow cytometry assay, we found a significant decrease in neutrophil survival upon neutralizing TNF‐α or IL‐8 (43.1 ± 3%, 51.2 ± 1.6%, respectively) compared with a2NuΦ control (57.9 ± 2.7%). Neutralizing both TNF‐α and IL‐8 together showed a more potent decrease in the percentage of neutrophil survival (37.6 ± 2.3%). We confirmed these results by quantifying the expression of the active caspase‐3. Consistent results were obtained, as the percentage of apoptotic cells increased upon neutralizing TNF‐α or IL‐8 (40.9 ± 2%, 36.5 ± 1.9%, respectively), and this percentage further increased upon neutralizing both TNF‐α and IL‐8 together (45.5 ± 1.4%) in comparison with the a2NuΦ control (28.9 ± 1.5%). In addition, we checked the effect of a2NTD treatment on growth factor expression in neutrophils. There was an elevated mRNA expression of G‐CSF in a2NuΦ, and that expression was dependent on NF‐κB activation (Fig. S5A). We confirmed that by quantifying the protein levels of G‐CSF and GM‐CSF. There was a significant increase in G‐CSF and GM‐CSF secreted levels in comparison with control. NF‐κB inhibition decreased the levels of G‐CSF but not GM‐CSF (Fig. S5B,C). Neutralizing G‐CSF by anti‐G‐CSF did not change the percentage of the apoptotic cells compared with a2NuΦ control (Fig. S5D–F). Together, these data confirm that a2NTD treatment stimulates the secretion of prosurvival cytokines, such as TNF‐α and IL‐8 from neutrophils, that act in an autocrine manner and support neutrophil survival.

### 3.7 TLR2 blockade partially inhibits the a2NTD effect on neutrophil survival

**Figure 7:**
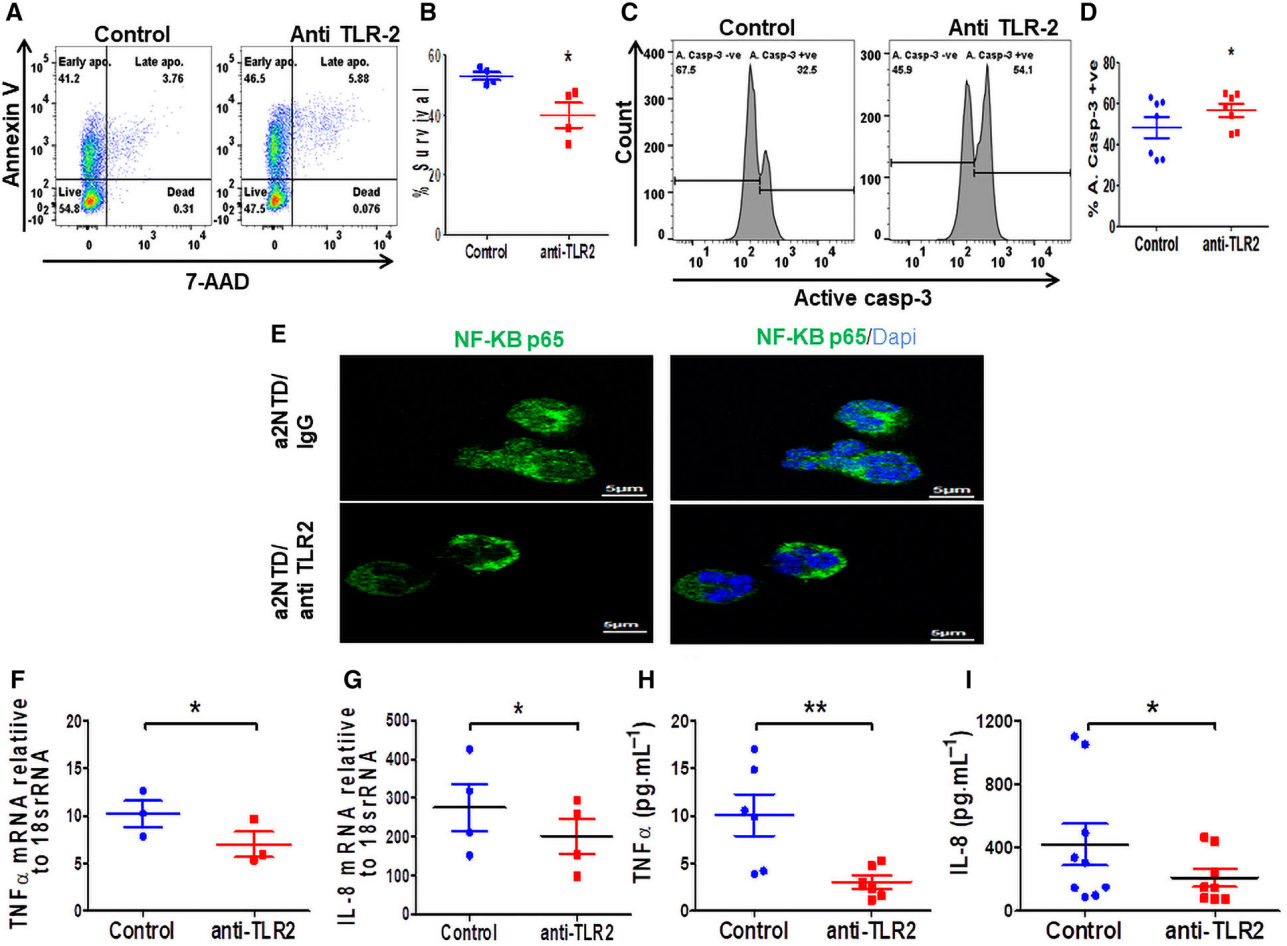
Blocking TLR2 retards partially the a2NTD effect on neutrophil survival. (A, B) Assessment of neutrophil apoptosis at 18‐h incubation after blocking TLR2 on neutrophils by anti‐TLR2 blocking antibody or IgG (10 μg·mL^−1^) as a control followed by stimulation with a2NTD. Results are shown as mean ± SEM (*n* = 4). Statistical significance of group differences determined by two‐tailed paired Student’s *t*‐test, *P < 0.05. (C, D) Active caspase‐3 expression in neutrophils after 18‐h stimulation by a2NTD in the presence of anti‐TLR2 or IgG (control). Data presented as mean ± SEM (*n* = 7). Statistical significance of group differences determined by two‐tailed paired Student’s *t*‐test, *P < 0.05. (E) NF‐κB p65 immunofluorescence staining in a2NTD‐treated neutrophils (a2NTD 500 ng·mL^−1^, 1 h) after blocking the cells with anti‐TLR2 or IgG (control). NF‐κB p65 (green), nucleus (DAPI, blue). Images were taken by confocal microscopy, scale bars: 5 μm. Representative images are presented from 3 different experiments. (F, G) mRNA gene expression of TNF‐α and IL‐8 in a2NTD‐treated neutrophils (a2NTD 500 ng·mL^−1^, 4 h) in the presence of anti‐TLR2 or IgG (control). Plots represent data as mean ± SEM (*n* = at least 3). Statistical significance of group differences determined by two‐tailed Mann–Whitney test, *P < 0.05. (H, I) Secreted levels of TNF‐α and IL‐8 from a2NTD‐treated neutrophils (a2NTD, 500 ng·mL^−1^, 18 h) after blocking with anti‐TLR2 or IgG. Results presented as mean ± SEM (*n* = at least 6), Statistical significance of group differences determined by two‐tailed paired Student’s *t*‐test, *P < 0.05, **P < 0.01.

TLR2 activation by proteins and peptides stimulates ROS generation, cytokine production, and NF‐κB activation (Prince *et al.*, 2011). These findings let us investigate whether a2NTD acts by binding and activation of TLR2. Blocking of TLR2 by specific antibody leads to a significant decrease in the percent survival of a2NuΦ relative to control antibody‐treated cells (Fig. 7A,B). Also, the percentage of the apoptotic cells increased upon TLR2 receptor blockade (Fig. 7C,D). Furthermore, immunofluorescence analysis demonstrated the decrease in the NF‐κB p65 translocation to the nucleus after blocking TLR2 receptor (Fig. 7E). Confirming this finding, we checked the gene expression (Fig. 7F,G) and the protein levels (Fig. 7H,I) of TNF‐α and IL‐8 in TLR2‐blocked a2NuΦ. We found a significant decrease in the mRNA and protein levels of both TNF‐α and IL‐8 production upon the blockage of TLR2. These results suggest that a2NTD can promote neutrophil survival by binding to TLR2 receptor.

### 3.8 Effect of a2NTD treatment on the extrinsic pathway of apoptosis in neutrophils

**Figure 8:**
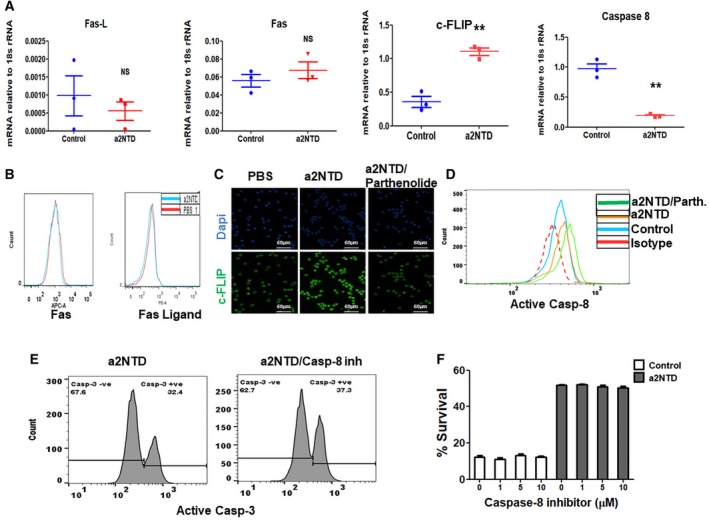
Effect of a2NTD treatment on the extrinsic pathway of apoptosis in neutrophils. (A) Quantitative real‐time PCR was performed to assess the mRNA gene expression of Fas, Fas ligand, c‐FLIP, and caspase‐8. Data plotted as mean ± SEM (*n* = at least 3). Statistical significance of group differences determined by two‐tailed Mann–Whitney test, **P < 0.01. (B) Flow cytometry analysis of the surface expression of Fas and Fas ligand on neutrophils. (C) Immunofluorescence staining of c‐FLIP in neutrophils after treatment with PBS (control), and a2NTD in the presence or absence of parthenolide (2.5 μm) for 4 h. Representative images of 3 different experiments, c‐FLIP (green), DAPI (blue). Imaging was done by fluorescence microscope, original magnification 400×, scale bars: 50 μm. (D) Active caspase‐8 expression in neutrophils was measured by flow cytometry. Neutrophils were treated by PBS (control) and a2NTD with or without parthenolide for 18 h. Representative histogram from 3 different experiments. (E, F) Assessment of the effect of caspase‐8 inhibitor on a2NTD‐treated neutrophil apoptosis by staining for active caspase‐3 or using Annexin‐V/7‐AAD assay, respectively. Data presented as mean ± SEM (*n* = 3). No statistical significance of group differences determined by two‐tailed paired Student’s *t*‐test.

We also investigated the effect of a2NTD on the extrinsic pathway of apoptosis in neutrophils. We found that a2NTD treatment did not cause a change in the mRNA or the protein expression of Fas or Fas ligand (FasL) (Fig. 8A,B). However, the mRNA and the protein levels of c‐FLIP, cellular FLICE (FADD‐like IL‐1β‐converting enzyme) inhibitory protein, an important antiapoptotic regulator, were substantially increased in a2NuΦ (Fig. 8A,C). Also, caspase‐8 mRNA levels in neutrophils decreased upon a2NTD treatment (Fig. 8A), but there was a slight increase in active caspase‐8 level (Fig. 8D). To investigate the role of caspase‐8 in regulating a2NuΦ survival, we used caspase‐8 inhibitor. Caspase‐8 inhibitors did not change the percentage survival or the percentage of the apoptotic cells of a2NuΦ (Fig. 8E,F). This suggests that the changes that occurred in the extrinsic pathway of apoptosis in neutrophils upon a2NTD treatment did not participate in the prosurvival effect of a2NTD on neutrophils.

## 4 Discussion

In this study, we demonstrate a novel role of the cancer‐associated a2 isoform vacuolar ATPase (a2V) in regulating innate immunity. We provide evidence that a unique cancer‐derived peptide (a2NTD) promotes neutrophil survival. Many studies report that high levels of TAN are associated with poor outcomes in various types of cancer including renal cell carcinoma and bronchoalveolar cancer (Jensen *et al.*, 2009; Wislez *et al.*, 2003). Recent study by Gentles *et al* performed a transcriptome computational analysis that demonstrates that TANs are the most adverse prognostic cell population in over 3000 solid tumors including 14 cancer type tumor (Gentles *et al.*, 2015). In agreement, we found that an increased neutrophil numbers in cancer tissues are associated with aggressive tumors based on the state of differentiation of cancer tissues.

TANs survive longer than circulating neutrophils that allow TAN to promote tumor growth and metastasis (Fridlender and Albelda, 2012; Pylaeva *et al.*, 2016; Queen *et al.*, 2005). Studies suggest that TANs have long life span due to chemokines and growth factor presence in the TME (Clappaert *et al.*, 2018; Sawanobori *et al.*, 2008). These factors are hard to target because they are not specific to the cancer microenvironment but also play important roles at normal physiological conditions or at host defense against pathogens. Defining targets specific to cancer microenvironment will lead to the production of precise specific cancer therapy. Our study supports the hypothesis that the origin of TAN could be the peripheral neutrophils recruited to site of the tumor where tumor‐secreted factors reprogram the neutrophil function to support cancer progression. Additionally, the presence of multiple forms of neutrophils such as TAN, low‐density neutrophils (LDN), and myeloid‐derived suppressor cells (MDSCs) could be due to the effect of tumor‐secreted factors that stimulate neutrophil differential exocytosis of distinct granules that leads to modulation of neutrophil phenotype, surface markers, and density. Overall, these changes play an important role to promote cancer growth, metastasis, and immunosuppression (Mollinedo, 2019).

V‐ATPases are multisubunit proton pumps, highly expressed on cancer cells, and regulate cancer growth, metastasis, immune response, and drug resistance (Marshansky *et al.*, 2014; Stransky *et al.*, 2016). Targeting certain subunits in the V‐ATPases demonstrates a high potential for cancer treatment (Stransky *et al.*, 2016; Whitton *et al.*, 2018). We showed earlier that the a2 isoform of the ‘a’ subunit present in the V0 transmembrane domain is highly expressed in invasive breast cancer and this expression was associated with increased neutrophil infiltration and increased angiogenesis (Ibrahim *et al.*, 2015). *In vivo* and *in vitro* studies showed that specific deletion of a2V from cancer cells impaired the tumor growth and metastasis (Katara *et al.*, 2015) and sensitized cancer cells to cancer therapy (Kulshrestha *et al.*, 2016). Here, we show that human cancer tissues from four different organs express increased levels of a2V in comparison with adjacent normal tissues. This elevated expression was positively correlated with neutrophil count that suggests that elevated expression of a2V can be a marker in cancer and regulates neutrophils in the tumor microenvironment.

a2NTD promotes the protumorigenic properties of neutrophils and results in increased angiogenesis and promoted cancer cell invasiveness (Ibrahim *et al.*, 2015) and acts as a chemotactic agent to neutrophils (Ibrahim *et al.*, 2016). Here, we report that a2NTD promotes neutrophil survival and involves ROS generation. ROS at certain levels exert oxidative stress and too much damage, which are toxic to the cells. The correct cellular response to ROS production is important in order to prevent further oxidative damage and to maintain cell survival. One of the important pathways that are modulated by ROS is the NF‐κB pathway. NF‐κB target genes would attenuate ROS to promote survival (Morgan and Liu, 2011). Those findings are consistent with our data that a2NTD treatment stimulates ROS generation that led to NF‐κB activation and delayed neutrophil apoptosis.

**Figure 9:**
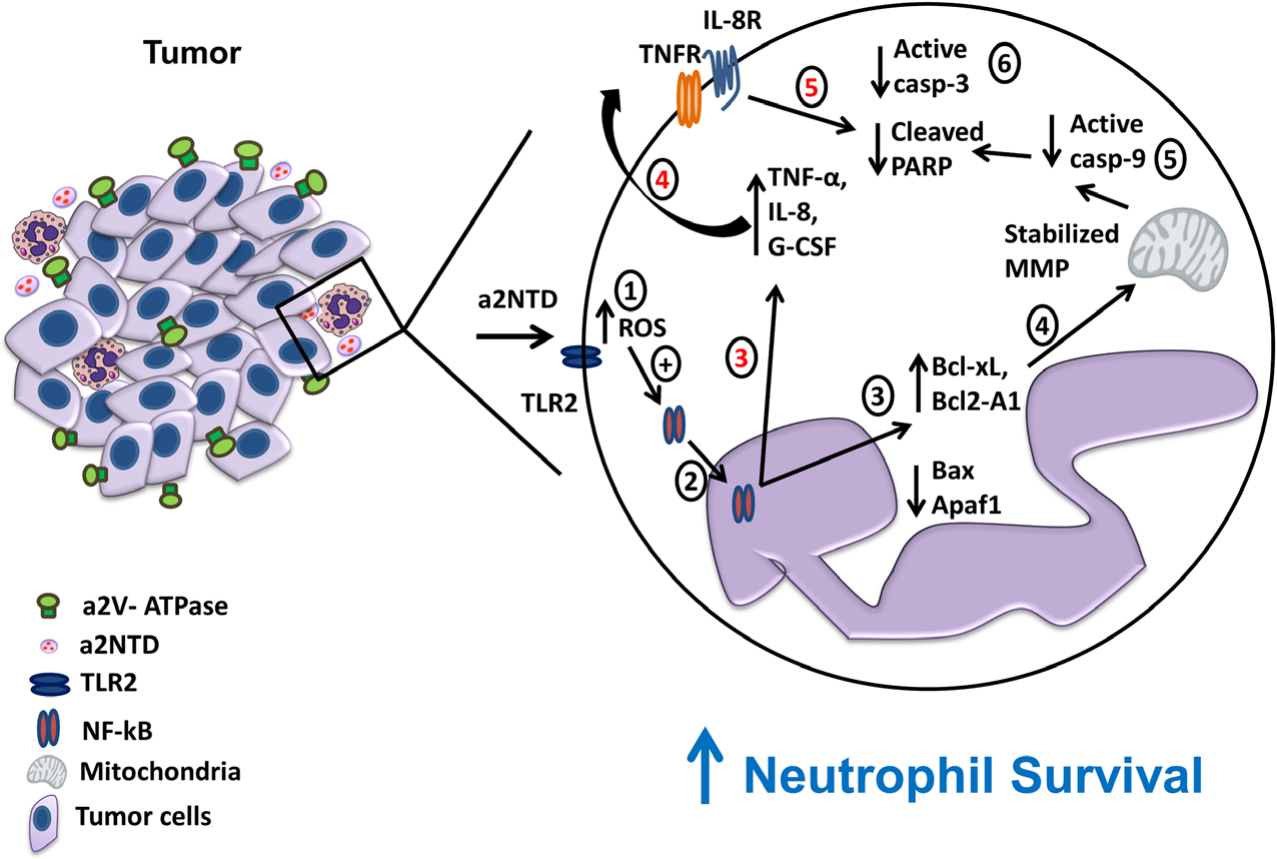
Schematic diagram summarizes the proposed mechanism by which a2V regulates neutrophil survival. a2V is highly expressed in cancer cells, and a2NTD is secreted to the TME and promotes neutrophil survival. a2NTD stimulates TLR2 receptor on neutrophils and ROS generation. ROS activates NF‐κB translocation to the nucleus. NF‐κB activation upregulates the expression of antiapoptotic proteins, Bcl‐2A1 and Bcl‐xL, and prosurvival cytokines, TNF‐a and IL‐8. This process was associated with a decrease in Bax and Apaf‐1 expression and a decrease in Bax translocation to the mitochondria that stabilizes the MMP and retains cyt‐*c* in the mitochondria and decreases caspase‐9 activation. The other arm of this mechanism is TNF‐α and IL‐8, they act in an autocrine manner and decrease neutrophil apoptosis by decreasing caspase‐3. MMP, mitochondrial membrane potential.

NF‐κB is a central mechanism that regulates inflammation and cell survival (Liu *et al.*, 2017). Several antiapoptotic members of the NF‐κB family are upregulated in TAN, but it is not clear whether this pathway is responsible for TAN longevity (Fridlender and Albelda, 2012; Fridlender *et al.*, 2012). We show that NF‐κB activation in neutrophils by a2NTD regulates the expression of Bcl‐2 family members and caspase expression and promotes the production and secretion of prosurvival cytokines and growth factors. The balance between the antiapoptotic and proapoptotic members of the Bcl‐2 family is important to decide cell fate (Geering and Simon, 2011). In neutrophils, the proapoptotic proteins including Bax are constitutively expressed and have long half‐life. However, the induction of short half‐life antiapoptotic proteins such as Bcl‐xL, Bcl2‐A1, and Mcl‐1 leads to a delay in neutrophil apoptosis (Akgul *et al.*, 2001; Geering and Simon, 2011). a2NTD treatment induced the Bcl‐xL and Bcl2‐A1 expression and reduced the expression of Bax in neutrophils. In the intrinsic apoptosis pathway, Bax translocates to the mitochondria and disturbs the outer membrane potential that leads to the leakage of cyt‐c to the cytoplasm to form the apoptosome with Apaf‐1 to cleave caspase‐9. a2NTD treatment hampers this pathway and significantly decreases caspase‐9 activation (Fig. 9).

Cytokines and growth factors including IL‐8, TNF‐α, G‐CSF, and GM‐CSF are known to prolong neutrophil survival and are also known as positive driver of tumor growth, angiogenesis, and metastasis (Elbim and Estaquier, 2010; Queen *et al.*, 2005). a2NTD treatment increased the production and the secretion of these mediators. NF‐κB inhibitor significantly decreased the levels of IL‐8, TNF‐α, and G‐CSF but not GM‐CSF, suggesting that a2NTD can also regulate other pathways. Blocking the effect of IL‐8 and/or TNF‐α by neutralizing antibodies increases apoptosis in a2NTD‐treated cells. IL‐8 and TNF‐α are inducers of NF‐κB activation (Manna and Ramesh, 2005), and they are NF‐κB target genes (Collart *et al.*, 1990; Kunsch and Rosen, 1993). a2NTD treatment activates NF‐κB, and this activation stimulates the secretion of IL‐8 and TNF‐α that can continuously activate NF‐κB and maintain neutrophil survival (Fig. 9). To our knowledge, this is the first report that shows the impact of a positive feedback loop between NF‐κB activation and the autocrine secretion of IL‐8 and TNF‐α on neutrophil survival by a cancer‐associated peptide.

TLR2 detects danger signals and activates NF‐κB and cytokine production (Prince *et al.*, 2011; Scheibner *et al.*, 2006). Several proteins and peptides including high mobility group box 1 (HMGB1), heat‐shock proteins (HSPs), and versican released in the TME activate myeloid cells through TLR2 to induce inflammatory TME and stimulate metastasis(Kim and Karin, 2011; Kim *et al.*, 2009; Shi *et al.*, 2016; West *et al.*, 2017). Tessarolli *et al* showed that TLR2 absence influences neutrophil survival (Tessarolli *et al.*, 2010). In fact, we found that blockage of TLR2 using blocking antibody partially negated the effect of a2NTD on neutrophil survival and led to decreased activation of NF‐κB and its target proteins TNF‐α and IL‐8. These data suggest that one way that a2NTD exerts its action is by the stimulation of TLR2. Further investigations are warranted to clarify our understanding of a2NTD binding receptors and downstream mechanisms.

## 5 Conclusion

This study demonstrates the important role of the V‐ATPases in cancer microenvironment and demonstrates a new link between cancer‐associated a2V and innate immunity. There is an important need to identify cancer‐associated proteins that could be effectively targeted by cancer therapeutics. This study suggests that high levels of a2V are a characteristic of cancer cells compared with their respective normal tissues and this high level of a2V is important for immune regulation. We demonstrate the mechanism that cancer‐associated a2V supports neutrophil survival by the action of its unique peptide a2NTD. Together with our previous studies, these studies suggest that a2V is a potential target for cancer treatment.

## Conflict of interest

The authors declare no conflict of interest.

## Author contributions

SAI conceived, designed, and performed experiments, interpreted the data, undertook statistical analyses, and wrote the manuscript. AK participated in data organization and was involved in drafting the manuscript. VR assisted in RNA isolation. GKK and MR provided intellectual support. KDB contributed to the study design, data interpretation, and manuscript preparation and provided funds for the study. All authors read and approved the final manuscript.

## Supporting information


**Fig. S1.** a2NTD specific effect on neutrophil survival and on caspase expression.
**Fig. S2.** Time course of neutrophil survival, apoptosis and active caspase‐3 at different doses of a2NTD treatment.
**Fig. S3.** a2NTD effect on ROS generation and NF‐κB activation in neutrophils.
**Fig. S4.** Effect of NF‐κB inhibition on apoptosis related protein expression in a2NTD treated neutrophils.
**Fig. S5.** Effect of a2NTD treatment on G‐CSF secretion from neutrophils.
**Fig. S6.** Assessment of the neutrophil mitochondrial membrane potential (MMP).
**Fig. S7.** Specific effect of a2NTD on neutrophils.
