# Supplementary material for: Cancer‐associated V‐ATPase induces delayed apoptosis of protumorigenic neutrophils
Source: Mol Oncol. 2020 Jan 31;14(3):590–610. doi: 10.1002/1878-0261.12630 (PMC7053242; doi:10.1002/1878-0261.12630)
Supplement: Supplementary file 1 — Fig. S1. a2NTD specific effect on neutrophil survival and on caspase expression. Fig. S2. Time course of neutrophil survival, apoptosis and active caspase‐3 at different doses of a2NTD treatment. Fig. S3. a2NTD effect on ROS generation and NF‐κB activation in neutrophils. Fig. S4. Effect of NF‐κB inhibition on apoptosis related protein expression in a2NTD treated neutrophils. Fig. S5. Effect of a2NTD treatment on G‐CSF secretion from neutrophils. Fig. S6. Assessment of the neutrophil mitochondrial membrane potential (MMP). Fig. S7. Specific effect of a2NTD on neutrophils. [file MOL2-14-590-s001.docx]

**Supporting information:**

**
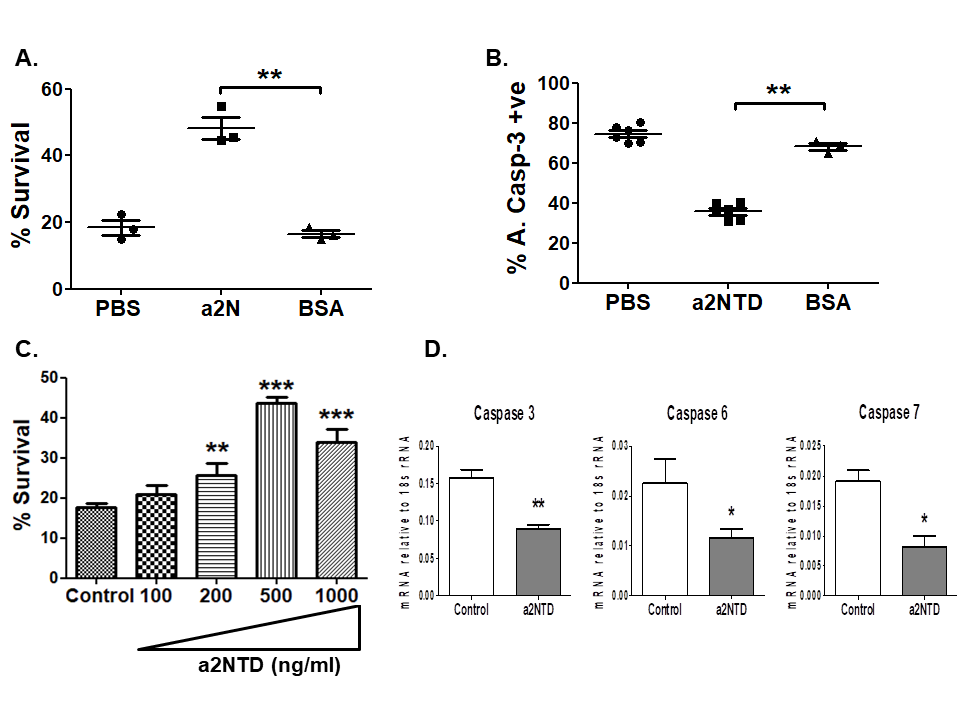
**

**Supplemental Figure S1: a2NTD specific effect on neutrophil survival and on caspase expression.**

**A)** Quantification of the percentage survival of (Annexin-V^-^/7-AAD^-^ cells) of neutrophils (n= 3), BSA; bovine serum albumin. **B)** Quantitative analysis of the percentage of the active caspase-3 positive cells using flow cytometry. Experiments were done in duplicate (n= at least 3). **C)** The effect of a2NTD on neutrophil survival in a concentration dependent manner (n= at least 5). Results presented as mean ± SEM, **P < 0.01, ***P < 0.001 as compared with PBS (control) treated neutrophils. **D)** Quantitative real time-PCR was performed to assess the mRNA gene expression of caspase-3, -6, and -7, in a2NTD or PBS treated neutrophils after 4h incubation. Data reported as mean of mRNA expression relative to 18s rRNA ± SEM from at least three different independent experiments each was done in triplicate. *P < 0.05, **P < 0.01 compared to the control neutrophils.


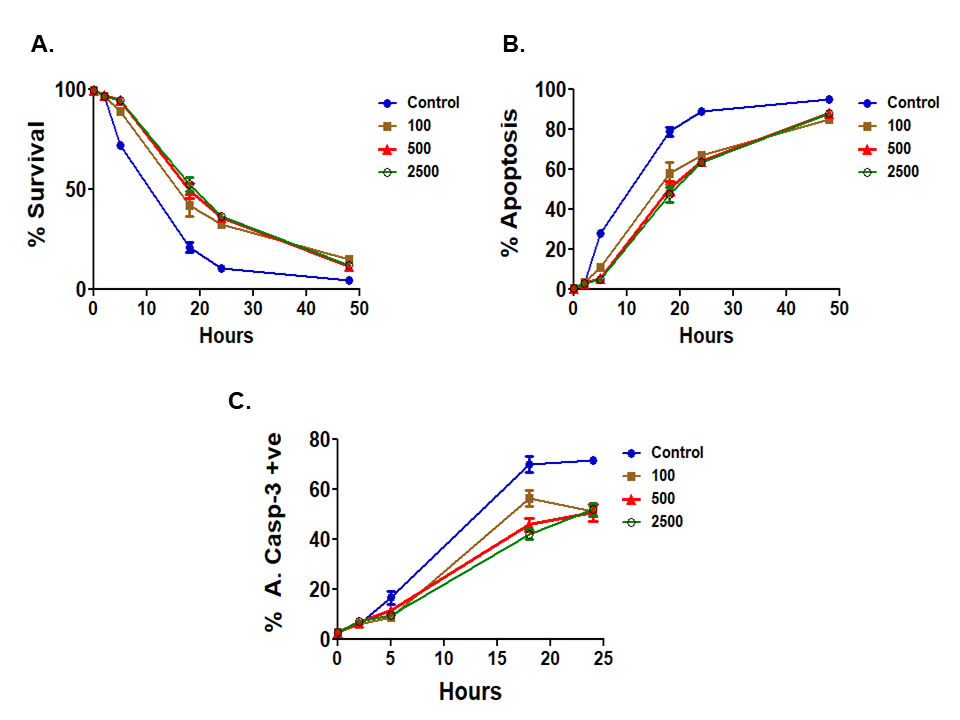


**Supplemental Figure S2: Time course of neutrophil survival, apoptosis and active caspase-3 at different doses of a2NTD treatment.**

Assessment of the viability of neutrophils as well as the active caspase-3 expression using flow cytometry, in at least 5 different time points (0h, 2h, 5h, 18h, 24h, 48h) after treatment with different concentrations of a2NTD (0, 100, 500, 2500 ng/ml). **A)** Quantification of the percentage survival of (Annexin-V^-^/7-AAD^-^ cells) of neutrophils, **B)** quantification of the percentage of the apoptotic cells (early and late apoptotic cells). Data reported as mean ± SEM (n= 3). **C)** Quantitative analysis of the percentage of the active caspase-3 positive cells using flow cytometry. Data is shown as mean ± SEM (n= 3).


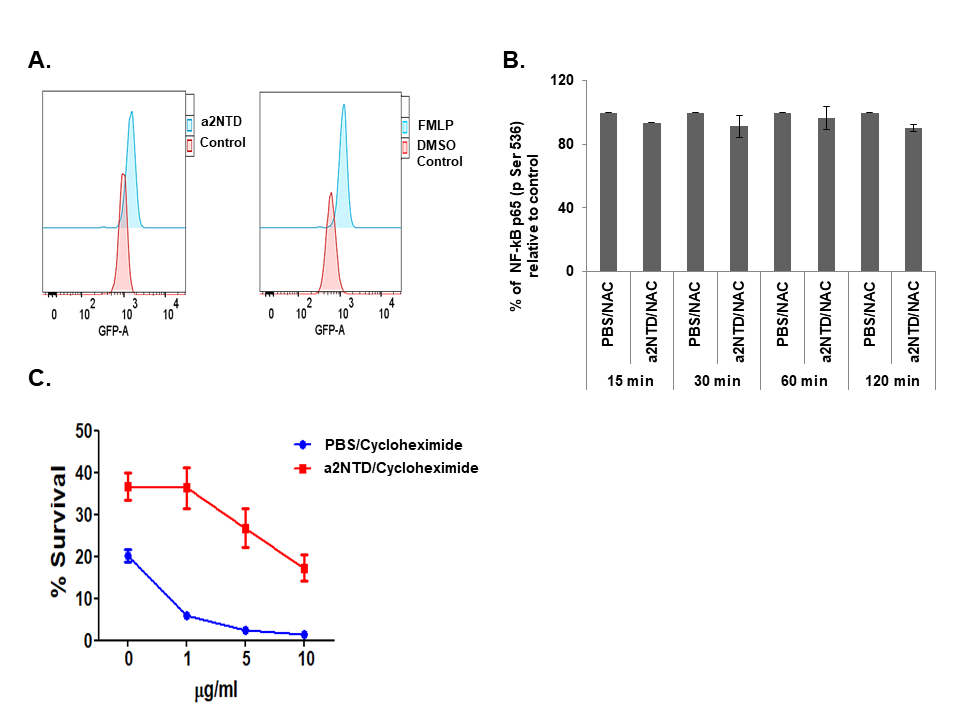


**Supplemental Figure S3: a2NTD effect on ROS generation and NF-κB activation in neutrophils.**

**A)** Flow cytometric analysis of ROS generation by control, a2NTD or FMLP (positive control) treated neutrophils using CM-H_2_DCFDA. Representative histograms from six independent experiments were done in duplicate. **B)** Quantification of NF-κB p65 phosphorylation at Ser 536 in neutrophil lysates by ELISA after different time point of stimulation by a2NTD (500 ng/ml) or PBS (control). Data were collected from at least 3 independent experiments and are reported as the mean of percentage increase of NF-κB p65 (pSer 536) relative to control ± SEM. **C)** Quantification of the percentage survival of (Annexin-V^-^/7-AAD^-^ cells) of neutrophils in control or a2NTD treated neutrophils after pretreatment with different concentration of cyclohexamide. Results presented as mean percentage survival ± SEM, (n= 3).


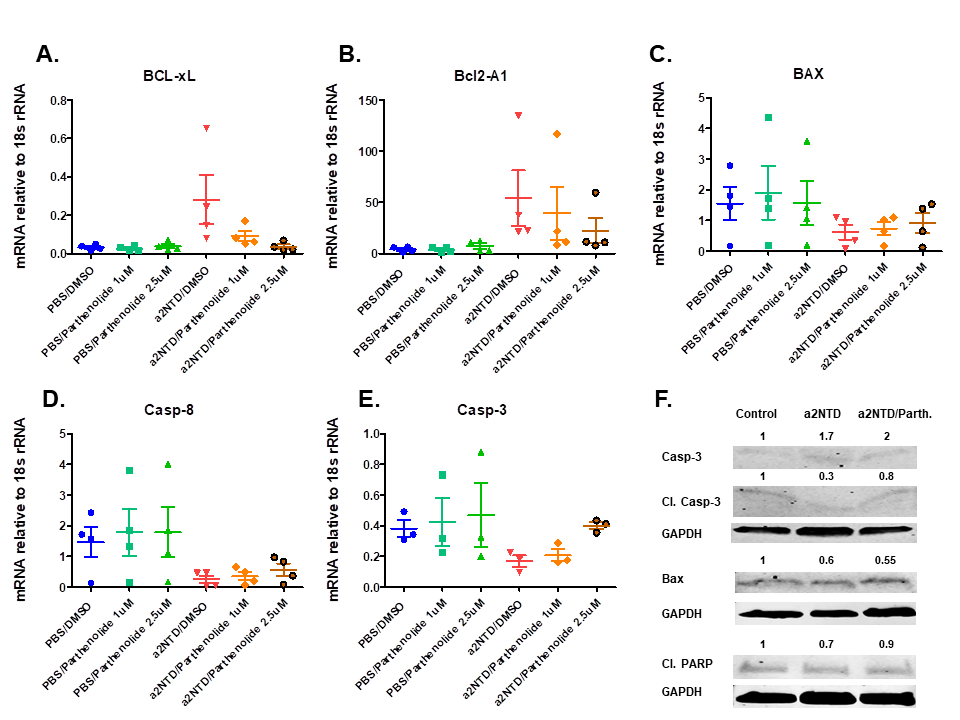


**Supplemental Figure S4: Effect of NF-κB inhibition on apoptosis related protein expression in a2NTD treated neutrophils.**

Quantitative real time-PCR was performed to assess the mRNA gene expression of A) Bcl-xL, B) Bcl2-A1, C) Bax, D) caspase-8 and E) caspase-8 in a2NTD or PBS treated neutrophils after 4h incubation in the presence of 1 μM or 2.5 μM parthenolide. Data reported as mean of mRNA expression relative to 18s rRNA ± SEM from at least three different independent experiments each was done in triplicate. **F)** Immunoblotting analysis of total casp-3, active casp-3, Bax, and cleaved PARP in neutrophil lysates treated with PBS (control), a2NTD, or a2NTD/parthenolide (2.5 μM) for 18 h incubation. Representative images of two individual experiments.


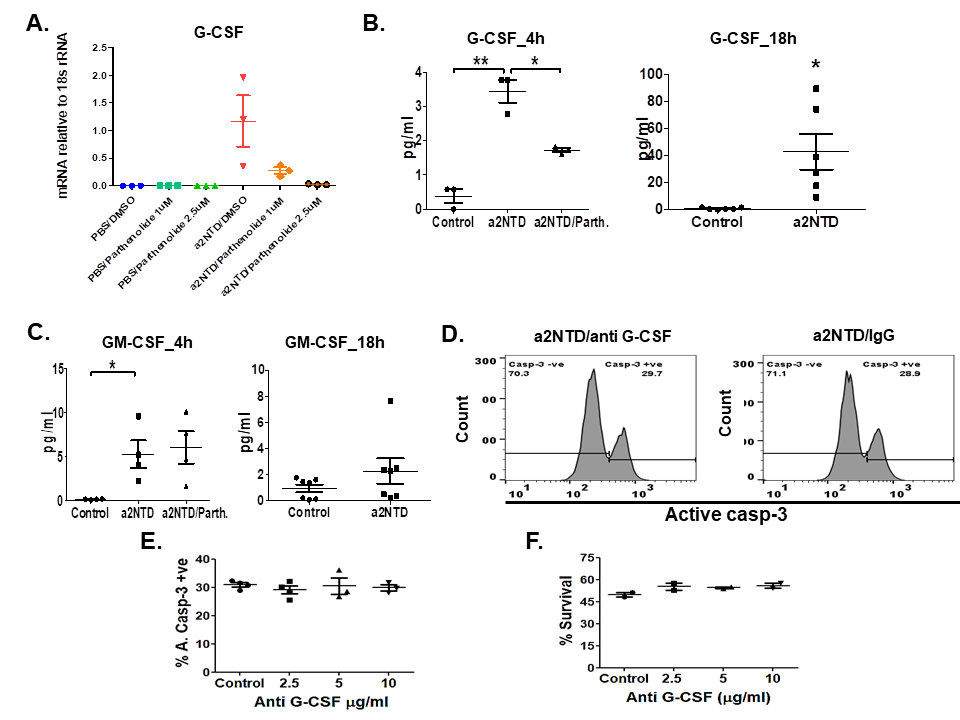


**Supplemental Figure S5: Effect of a2NTD treatment on G-CSF secretion from neutrophils.**

**A)** Quantitative real time-PCR was performed to assess the mRNA gene expression of G-CSF in a2NTD or PBS treated neutrophils after 4h incubation in the presence of 1 μM or 2.5 μM parthenolide. Data reported as mean of mRNA expression relative to 18s rRNA ± SEM from at least three different independent experiments each was done in triplicate. **B) & C)** Quantification of G-CSF & GM-CSF secreted protein levels (pg/ml) by multiplex luminex assay in neutrophil supernatant collected after 4 h incubation in presence or absence of parthenolide (2.5 μM) for 18 h incubation. Data presented as mean ± SEM (n = at least 3), *P < 0.05, **P < 0.01 compared to the control neutrophils. **D)** Representative flow cytometry analysis of active caspase-3 expressed by a2NTD treated neutrophils after pretreatment with 10 μg/ml anti G-CSF (left) or IgG (right). Quantitative analysis of the percentage of **E)** active caspase-3 positive cells (n=at least 3) and **F)** percentage survival (n= 2) of a2NTD treated neutrophils after the pretreatment with different concentrations of anti G-CSF. Experiments were done in duplicate.


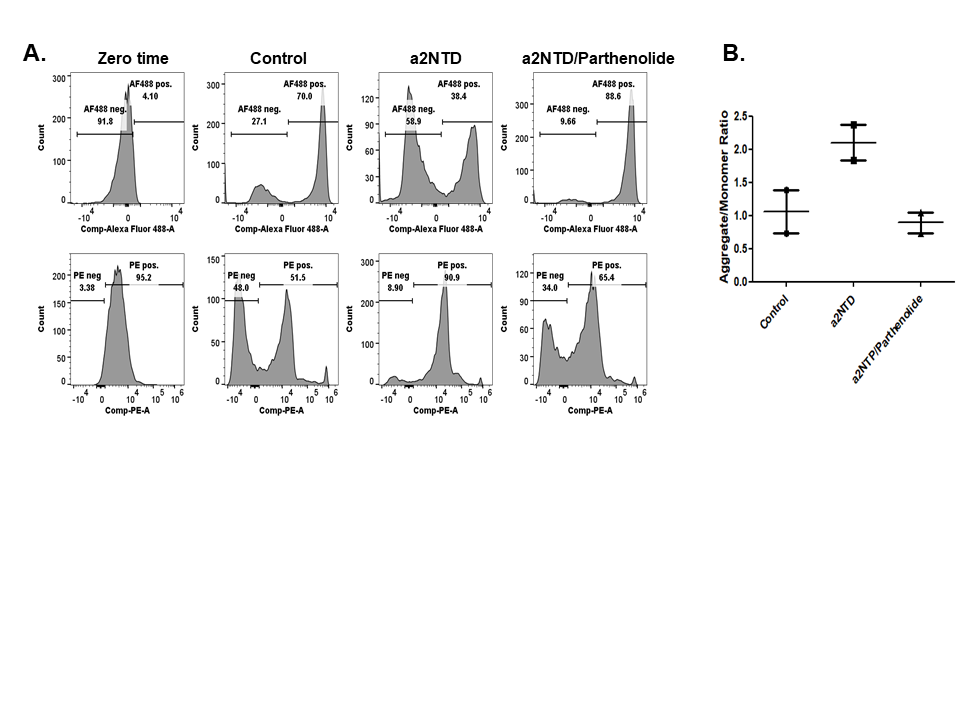


**Supplemental Figure S6: Assessment of the neutrophil mitochondrial membrane potential (MMP).**

**A)** Representative flow cytometric analysis of the neutrophil MMP using JC-1 dye at zero time and after 18h incubation. AF-488 positive cells represent cells having JC-1 monomer (disrupted MMP). PE positive cells represent cells having JC-1 aggregate (intact MMP). Control is PBS treated cells. **B)** Quantification of the aggregate/monomer ratio of JC-1 dye in neutrophils. Experiments were done in duplicate (n= 2).


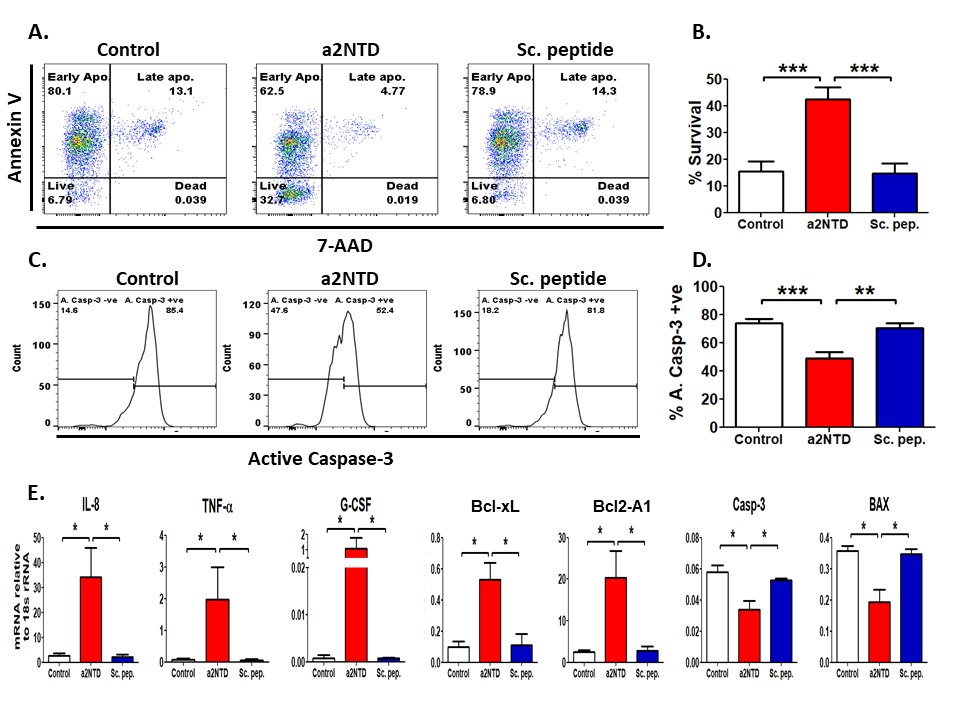


**Supplemental Figure S7:** Specific effect of a2NTD on neutrophils.

**A)** Representative dot plots of flow cytometry analysis to assess neutrophil apoptosis. Isolated neutrophils were treated with PBS (control), a2NTD 500ng/ml or scramble peptide (Sc. peptide) 500 ng/ml for 18 h incubation. **B)** Quantification of the percentage survival of (Annexin-V^-^/7-AAD^-^ cells) neutrophils. Experiments were done in triplicate (n=2). ***P < 0.001 as compared with control neutrophils or scramble peptide. **C)** Representative histograms showing the percentage of active caspase-3 expressed by control, a2NTD or scramble peptide (Sc. pep.) treated neutrophils. **D)** Quantitative analysis of the percentage of the active caspase-3 positive cells using flow cytometry. Experiments were done in triplicate (n=2). ***P < 0.001, **P < 0.01 as compared with control neutrophils or scramble peptide, respectively. **E)** Quantitative real time-PCR was performed to assess the mRNA expression of IL-8, TNF-a, G-CSF, Bcl-xL, Bcl2-A1, Casp-3 and Bax genes in control, a2NTD or scramble peptide neutrophils after 4h incubation. Data reported as mean of mRNA expression relative to 18s rRNA ± SEM from at three different independent experiments each was done in duplicate, *P < 0.05 as compared with control neutrophils or scramble peptide.
